# Sem1 links proteasome stability and specificity to multicellular development

**DOI:** 10.1371/journal.pgen.1007141

**Published:** 2018-02-05

**Authors:** Miriam Kolog Gulko, Gabriele Heinrich, Carina Gross, Blagovesta Popova, Oliver Valerius, Piotr Neumann, Ralf Ficner, Gerhard H. Braus

**Affiliations:** 1 Department of Molecular Microbiology & Genetics, University of Goettingen, Goettingen, Germany; 2 Goettingen Center for Molecular Biosciences (GZMB), University of Goettingen, Goettingen, Germany; 3 Center for Nanoscale Microscopy and Molecular Physiology of Brain (CNMPB), University of Goettingen, Goettingen, Germany; 4 Department of Structural Biology, University of Goettingen, Goettingen, Germany; Leibniz Institute for Natural Product Research and Infection Biology, GERMANY

## Abstract

The transition from vegetative growth to multicellular development represents an evolutionary hallmark linked to an oxidative stress signal and controlled protein degradation. We identified the Sem1 proteasome subunit, which connects stress response and cellular differentiation. The *sem1* gene encodes the fungal counterpart of the human Sem1 proteasome lid subunit and is essential for fungal cell differentiation and development. A *sem1* deletion strain of the filamentous fungus *Aspergillus nidulans* is able to grow vegetatively and expresses an elevated degree of 20S proteasomes with multiplied ATP-independent catalytic activity compared to wildtype. Oxidative stress induces increased transcription of the genes *sem1* and *rpn11* for the proteasomal deubiquitinating enzyme. Sem1 is required for stabilization of the Rpn11 deubiquitinating enzyme, incorporation of the ubiquitin receptor Rpn10 into the 19S regulatory particle and efficient 26S proteasome assembly. Sem1 maintains high cellular NADH levels, controls mitochondria integrity during stress and developmental transition.

## Author summary

The cellular ubiquitin-proteasome pathway is essential to control cell cycle, gene expression or the response to oxidative stress. Sem1 is conserved in eukaryotes from single cell yeasts to humans as intrinsically disordered and multifunctional protein. Sem1 supports the assembly of several multiprotein complexes but becomes eventually exclusively a subunit of the lid of the 26S proteasome, a cellular machine with a molecular mass of about two megadalton. Defects in the function of the proteasome, which degrades a large fraction of intracellular proteins, result in cancer or neurodegenerative diseases. We showed that Sem1 from a multicellular fungus is required for accurate 26S proteasome assembly and specific activity as prerequisites for mitochondria integrity, oxidative stress response and cell differentiation. Our findings of the complex and dynamic interplay between multiple cellular processes mediated by a small conserved intrinsically unordered protein sheds light and supports current efforts to understand and explore in more details potential therapies to eventually treat age-related human diseases.

## Introduction

The 26S proteasome represents the major cytoplasmic and nuclear ubiquitin-dependent protein degradation machinery and is composed of a barrel-like 2.5 MDa 20S proteolytic core particle (CP) capped with one or two 19S regulatory particles (RP). Proteins destined for degradation are unfolded, de-ubiquitinated and translocated by the RP to the CP where proteolytic activity takes place. Each regulatory particle consists of a lid and a base. The lid is composed of a nine-subunit protein composed of six PCI (Proteasome/COP9/Initiation factor) domain proteins (Rpn3, 5, 7, 9, 12), two MPN (Mpr1p and Pad1p N-terminal) domain proteins (Rpn 8, 11) and the Sem1 (Suppressor of Exocytosis Mutation 1) / Dss1 (deletion of split hand/split foot 1) protein. Sem1 was originally identified in *Saccharomyces cerevisiae* in genetic suppression studies of the exocyst [1]. It is a multifunctional and intrinsically disordered protein, associating with several functionally diverse protein complexes where it is also supporting assembly without being a subunit of the final active complex itself [2]. This includes the human BRCA2 complex (breast cancer complex) required for homologous recombination, the TREX2 complex (transcription export complex 2) needed for mRNA elongation and nuclear export, or the yeast Csn12-Thp3 complex involved in RNA splicing [2].

Deletion of the Sem1 encoding gene in yeasts resulted in viable cells with phenotypes including temperature sensitivity, enhanced filamentation and cell cycle delay [3]. Dss1 represents the human homolog and was able to rescue the growth defect of both *S*. *cerevisiae* and *S*. *pombe* mutant strains, suggesting a conserved molecular function from yeast to humans [4, 5].

The *in vivo* function of Sem1 in multicellular metazoans is difficult to address, as deletion of the corresponding gene in *C*. *elegans* revealed essential functions in oogenesis resulting in embryonic lethality and larval arrest [6]. The fungus *Aspergillus nidulans* can be used as model system as it not only grows by forming polar reiterated cellular units but can also differentiate and produce fruiting bodies including specialized cell types, which are surrounded by a tissue of auxiliary cells for nursing [7, 8].

The function of the fungal Sem1 counterpart was analysed in *A*. *nidulans*. The encoding gene was named here for convenience *sem1* and corresponds to *semA* according to *A*. *nidulans* nomenclature. Fungal Sem1 protein is associated with the 19S RP and links an appropriate oxidative stress response to cellular differentiation and coordinated fungal development. The majority of the proteasomes in the *Δsem1* mutant strain were 20S core particles, which provides an increased ATP-independent protease activity. The small proportion of cellular *Δsem1* deficient 19S and 26S proteasomes lacked any interaction with the chaperon Ecm29, which facilitates the association of the 19S RP with the 20S CP. The *Δsem1* 19S regulatory particles were also deprived from detectable incorporation of the ubiquitin receptor Rpn10, which facilitates the association of the lid with the base. The cellular redox state in *A*. *nidulans* is linked to Sem1-dependent transition from vegetative growth to differentiation. Sem1 links increased 26S proteasome stability to mitochondria integrity and is a prerequisite for an appropriate oxidative stress response required for multicellular development.

## Results

### Sem1 of the multicellular fungus *A*. *nidulans* is required for the formation of mature cleistothecia with meiotic ascospores

Sem1 proteins are conserved throughout eukaryotes from fungi to humans (S1 Fig). The impact of Sem1 on developmental programmes of a multicellular fungus was analysed. Filamentous fungi perform the transition from vegetative growth as hyphae to multicellular development by forming fruiting bodies consisting of tissues with distinct specialized cells. *A*. *nidulans* exhibits an asexual and a sexual life cycle propagated by spores. Light promotes asexual development and reduces sexual development, whereas darkness and oxygen limitation promote the sexual life cycle [9].

**Fig 1:**
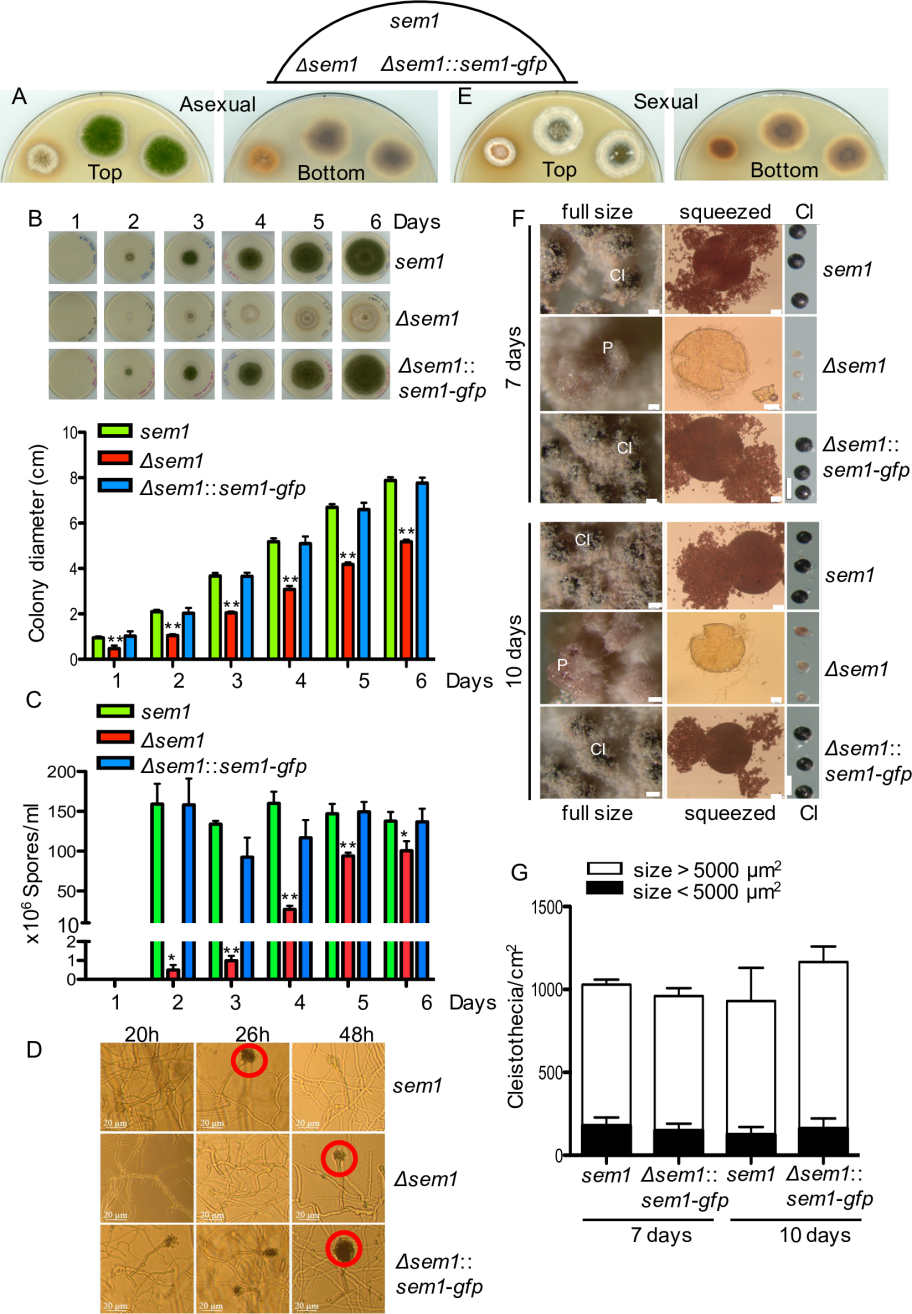
Sem1 is required for the maturation of cleistothecia in *A*. *nidulans*. **(A)** The *Δsem1* strain is delayed in formation of green asexual spores on minimal media in light in comparison to *A*. *nidulans* wildtype strain (*sem1*) or the complementation strain (*Δsem1*::*sem1-gfp*) with a functional Sem1-GFP. **(B)** *Δsem1* grows slower compared to *sem1* or complementation strains. Upper panel- the respective strains were point-inoculated with 10,000 spores and incubated in light for the indicated time points. Lower panel- the colony diameter was plotted. The wildtype grows 1.3±0.03 cm per day and *Δsem1* grows 0.81±0.03 cm per day. Significance of differences was calculated with t-test compared to *sem1*, **p<0.01, n = 4. **(C)** Deletion of *sem1* impairs asexual spore formation. Spores were visible in all strains only after 2 days of asexual growth. Wildtype and complementation produced ≈ 150x10^6^ spores/ml, this number remained similar over the time course. Increased conidia were measured in the deletion strain over time, with maxima of ≈ 94x10^6^ spores/ml after 5 days of asexual growth (64% compared to wildtype). For the quantification of asexual spore, plates were inoculated with 10,000 spores and harvested at the indicated time points. The values shown are the average ± SD from 2 independent experiments. Significance of differences was calculated with t-test compared to *sem1*, *p<0.05, **p<0.01. **(D)** *Δsem1* mutant strain shows delayed formation of conidiophores (red circles). All asexual growth impairments were restored in the complementation strain. **(E)** Sem1 is required for maturation of sexual fruiting bodies (cleistothecia) in darkness. **(F)** *Δsem1* mutant strain is block in early sexual development and develop only Hülle cells and primordia (p) after 10 days of sexual growth. Wildtype and complementation strains produced pigmented cleistothecia (Cl) after 7 days. Cleistothecia were squeezed to check for the presence of ascospores. Scale bars: 50 μm for full size images, 20 μm for squeezed cleistothecia and 200 μm for the panel comparing the size of the sexual structures in the respective strains. **(G)** Similar numbers of cleistothecia per cm^2^ were observed in wildtype or complementation strains. The average size of the cleistothecia from *Δsem1* was 5850±823 μm^2^. 15% of cleistothecia from wildtype and complementation had similar size and contained ascospores (Fig 1F, squeezed panel). Columns represent average number of cleistothecia ± SD per cm^2^, n = 2.

Deletion of the gene encoding fungal Sem1 resulted in a viable *Δsem1* deletion strain, which exhibited a reduced growth rate with a smaller colony size compared to wildtype (Fig 1A and 1B). The mutant strain accumulated a reddish pigment similar to a fungal strain defective in the COP9 signalosome, required for specific ubiquitination of substrates (Fig 1A and 1B) [10, 11]. Conidia are the asexual spores of *A*. *nidulans* and are formed at conidiophores. Conidia formation, which is normally favoured in light, is delayed in the absence of Sem1 (Fig 1C). Conidia formation was examined during 6 days of asexual growth. In the absence of Sem1, significant reduction of conidia was observed compared to wildtype and complementation strains. The wildtype and complementation strain produced ≈ 150x10^6^ spores/ml after only 2 days, whereas the mutant strain was able to produce less than 1% after 2 days (5000 spores/ml). The number of spores in the mutant strain increased over time, reaching a maximum after 5 days with approximately 64% of the spores produced by wildtype or complementation strains (Fig 1C). To determine whether the delay in conidiophore formation can explain the reduced conidia in the absence of Sem1, the morphology of conidiophores was examined after 20h, 26h and 48h (Fig 1D). No conidiation was observed in the absence of Sem1 after 20h. After 26h, most of the conidiophores of wildtype and complementation strains showed several lines of conidia, whereas the deletion strain only produced metulae and phialides. After 48h, all strains were able to produce conidiophores (Fig 1D). A gene encoding endogenously tagged Sem1-GFP complements these developmental phenotypes resulting in a functional gene that can be used to investigate *in vivo* Sem1 localization and interaction partners.

Growth of the deletion strain was examined in the dark, which promotes the formation of sexual fruiting bodies named cleistothecia, containing meiotic ascospores (Fig 1E and 1G). Cleistothecia maturation includes the formation of hyphal nests and primordia of fruiting bodies, a development that requires one week for wildtype or the *sem1-gfp* complemented strain. In contrast, the *Δsem1* strain showed after one week mostly white air mycelium and only nests with primordia (Fig 1F). Cleistothecia from wildtype and complementation strains contained meiotic ascospores, which were able to germinate, suggesting the ascospores are viable (Fig 1F, squeezed panel). Cultivation of *Δsem1* strain for 10 days still resulted in only white air mycelium and nests with primordia (Fig 1F, 10 days panel). Even after 10 days, cleistothecia from *Δsem1* strain contained only an empty cleistothecia-envelop without any ascospores, indicating a specific blockage in sexual fruiting body formation (Fig 1F, squeezed panel). The average size of these empty cleistothecia-envelops was 5850±823 μm^2^. In contrast, cleistothecia smaller than 5000 μm^2^ from wildtype or complementation strains (Fig 1G, 15%) were pigmented and contained ascospores (Fig 1F, squeezed panel).

These data demonstrate that the *Δsem1* mutant strain can grow vegetatively but is delayed in asexual development and blocked in sexual development with a misregulated secondary metabolism, as indicated by the accumulation of an orange dye. The finding that *sem1-gfp* can complement all these developmental phenotypes makes *A*. *nidulans* an attractive system to compare cellular 26S proteasome composition and assembly with or without Sem1 in a multicellular organism.

### Sem1 is required for efficient 26S proteasome assembly

**Fig 2:**
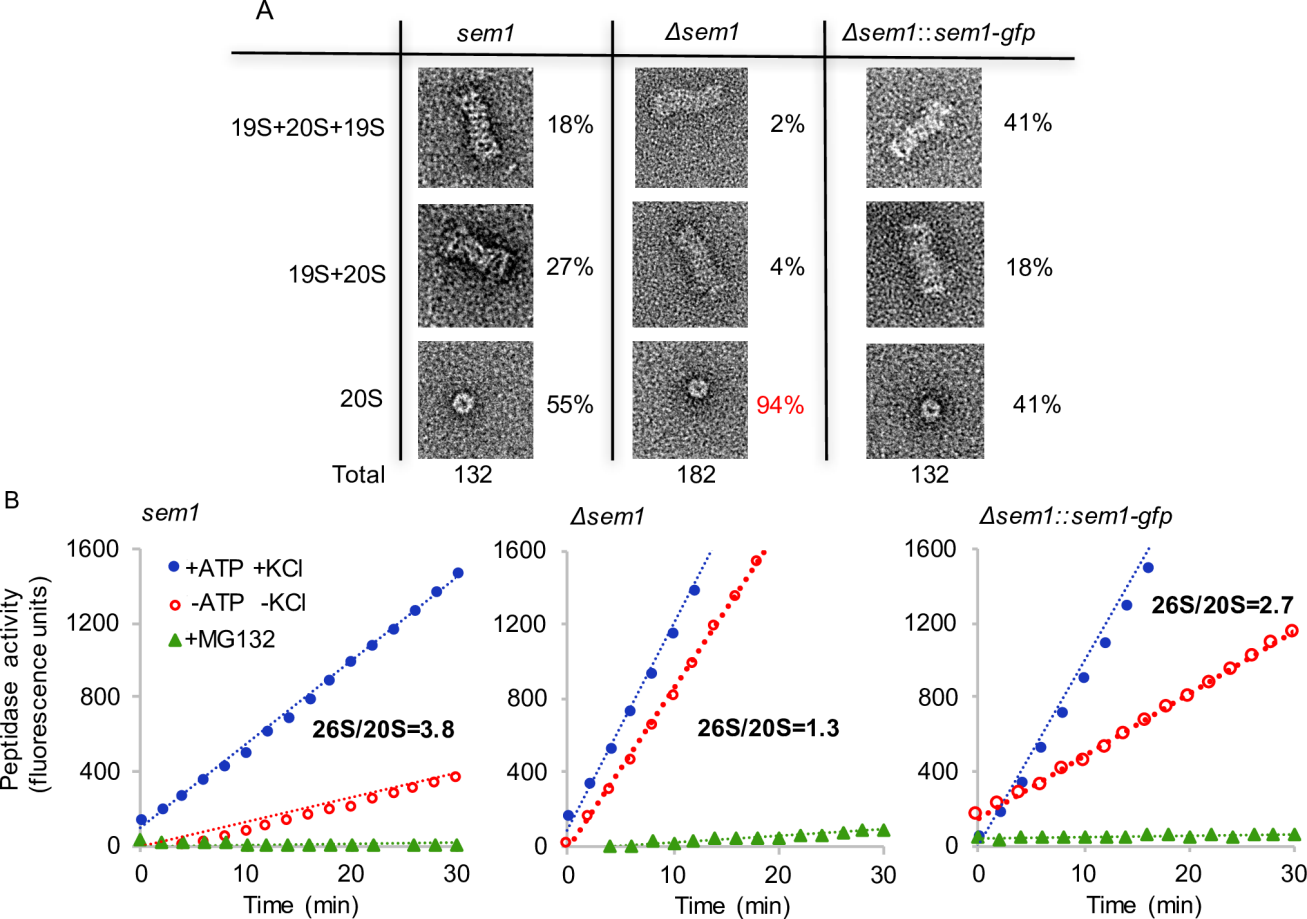
Accumulation of 20S proteasome complexes in *Δsem1*. **(A)** Electron micrographs of negatively stained proteasome complexes derived from fungal cell extracts. The total numbers of proteasome complexes and the respective complexes observed (%) in *Δsem1*, wildtype (*sem1*) or complemented (*Δsem1*::*sem1-gfp)* strains are indicated. **(B)** *Δsem1* proteasomes showed similar peptidase activities regardless to the presence or absence of ATP and KCl. Activities were measured in assay buffer containing 0.125mM ATP, 10mM KCl and 2.5μM of the proteasome inhibitor MG132. The assay buffer also contained 12.5 mM Tris HCl pH = 7.5 +1.25 mM MgCl_2_ +0.25 mM DTT+0.0125 mg/ml BSA. Blue, red and green curves represent peptidase activity of the indicated strains in the presence of ATP+KCl (26S activity), in the absence of ATP and KCl (-ATP–KCl, 20S) and in the presence of MG132 (proteasome inhibitor).

Cellular proteasome fractions from *Δsem1*, wildtype and *sem1-gfp* complementation strain were isolated and the ratios of intact 26S proteasomes versus 20S CP were compared. Negative staining electron microscopy revealed three forms of proteasome complexes in the cellular fractions, including a 20S CP, composed of a single capped (20S+19S) and a double-capped (19S+20S+19S) proteasomes, respectively (Fig 2A).

Cellular proteasomes with functional Sem1 comprise approximately half of the proteasomes as composed particles, ranging from 45% for Sem1 to 59% for Sem1-GFP. This includes 18% and 41% double-capped proteasomes for wildtype and Sem1-GFP complemented strain, respectively. Single capped proteasomes vary between 18% for the complemented and 27% for the wildtype strain in the analysed fungal extracts.

The ratio between assembled 26S and 20S proteasomes was significantly shifted within *Δsem1* mutant strain, which comprised only 6% composite proteasomes including only 2% double-capped proteasomes. Accordingly, the *Δsem1* mutant strain produced primarily 20S proteasome complexes (94%), whereas the 20S proteasome complexes of wildtype (55%) and Sem1-GFP complementation (41%) strains represent approximately half of the total cell extract derived proteasome fraction.

This increase in the percentage of 20S proteasomes from 50% to more than 90% of the total cellular proteasomes corroborates that Sem1 is required for an efficient *in vivo* assembly of 26S proteasomes and that Sem1-GFP can fulfil this function. The function of Sem1 might either be to accelerate the assembly or to stabilize functional 26S proteasomes or a combination of both. 20S CP might represent, even in the presence of Sem1, a substantial part of the cellular proteasome complexes during vegetative growth of the fungus.

### Proteasomes from *sem1*-deficient strain exhibit a significantly increased ATP-independent peptidase activity

Activities of purified proteasomes were measured by monitoring the hydrolysis of a fluorogenic peptide in the presence of ATP and KCl. *Δsem1* strain proteasomes showed 2.3 time higher rates of peptidase activities compared to the wildtype strain in the presence of ATP and KCl (Fig 2B, blue curves). Proteasome specificity was further demonstrated by the addition of the proteasome inhibitor MG132, which inhibited these peptidase activities almost completely to 3% or less (Fig 2B, green curves).

The overall proteasome activities in the presence of ATP were compared to the basal ATP-independent peptidase activities derived primarily from 20S CP in the absence of ATP and potassium ions (Fig 2B, red curves). These ATP-independent peptidase activities were lower than the overall activities of proteasomes in all strains, as the absence of ATP and potassium ions retard spontaneous activation of the 20S core particle [12]. In the presence of Sem1, the ATP-independent overall peptidase activity was 26% compared to ATP-dependent activity. This value increased to 75% in the *Δsem1* proteasome fraction. The ratio of 26S/20S peptidase activity in the wildtype and complementation strain was 3.8 times and 2.7 times higher, respectively, indicating that 26S proteasomes are more active than the 20S proteasomes. This ratio of peptidase activity was only 1.3 in *Δsem1* mutant strain, indicating similar peptidase activities in *Δsem1* regardless to the presence or absence of ATP and 19S RP. These data further support the finding of the electron microscopy analysis that Sem1 is required for the efficient assembly of functional 26S proteasomes. The high ATP-independent relative to ATP-dependent peptidase activity of *Δsem1* cells in comparison to wildtype could considerably contribute to the observed mutant phenotypes including the failure to establish developmental programmes.

### Sem1 supports the formation of ubiquitin-conjugated substrates and affects the ubiquitin conjugation pathway

**Fig 3:**
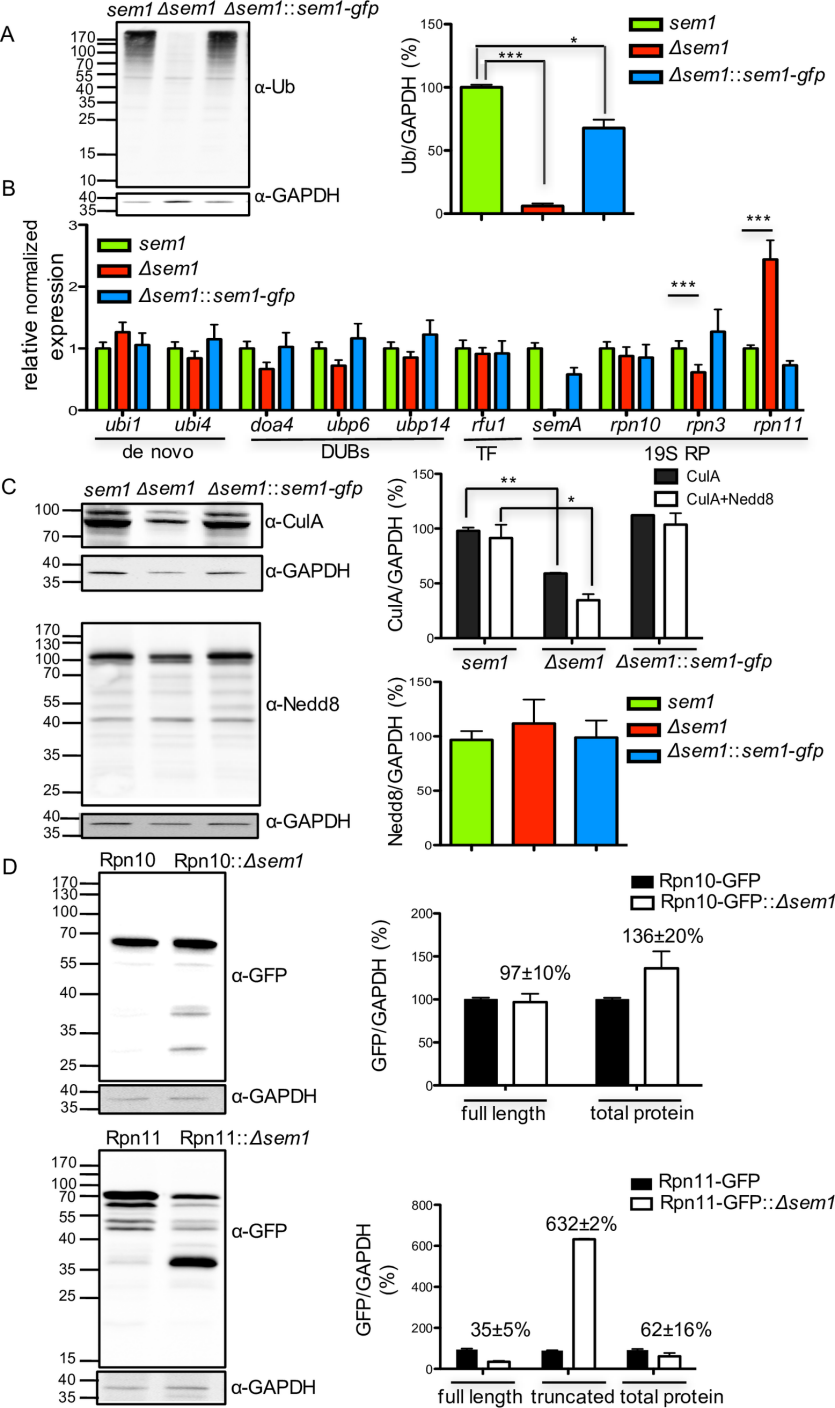
Deletion of *sem1* promotes decrease in the total ubiquitin-conjugates and destabilization of the Rpn11 deubiquitinating enzyme. **(A)** Significant decrease in polyubiquitylated substrates upon deleting *sem1*. Polyubiqutinated substrates were detected with α-ubiquitin and α-GAPDH served as loading control. T-test of *Δsem1* vs *sem1*, ***p<0.001 and *sem1* vs *Δsem1*::*sem1-gfp*, *p<0.05, n = 2. **(B)** Transcription levels of 19S RP subunits and genes involved in maintaining intracellular ubiquitin pools. RT-PCR results are shown as relative expression compared to *sem1*, n = 4, ***p<0.001. **(C)** Reduced amount of CulA/Cul1 protein in *Δsem1*. Neddylated substrates were detected with α-CulA (n = 3) and α-Nedd8 (n = 2), respectively, *p<0.05, **p<0.01. **(D)** Stability of Rpn10-GFP and Rpn11-GFP proteins in *Δsem1*. Western blots were probed with α-GFP to determine the relative amounts of full-length and truncated (37KDa) proteins. Mean intensities from three biological replicates were normalized to GAPDH (see also S2 and S3 Figs).

Significant changes in the ubiquitination pattern were detected in the *Δsem1* mutant strain with only 6% of conjugated substrates in *Δsem1* compared to wildtype cells (Fig 3A). This loss of ubiquitin conjugated proteins in the *Δsem1* mutant strain suggests a direct or indirect Sem1 effect on cellular ubiquitination process or on the control of components of the ubiquitin conjugation pathway.

Sem1-dependent transcription of six genes providing cellular ubiquitin was examined to analyse whether Sem1 affects cellular ubiquitin homeostasis. The *ubi1* gene encodes a protein where ubiquitin is fused to the small ribosome subunit; thereby synthesis of fusion protein will yield ribosomal protein and ubiquitin. The *ubi4* gene product contains four head to tail repeats of ubiquitin and supplies monoubiquitin to the cell. RT-PCR revealed that the transcription levels of both *de novo* synthesis genes of ubiquitin were similar in *Δsem1* mutant strain compared to wildtype (Fig 3B). The genes *doa4*, *ubp6* and *ubp14* encode deubiquitinating enzymes (DUBs), which recycle polyubiquitin chains. The *rfu1* gene encodes an inhibitor of Doa4 and balances the amount of monoubiquitin and polyubiquitin chains [13]. Transcript levels of these four genes involved in recycling of polyubiquitin were comparable in strains with or without functional Sem1 (Fig 3B). These data suggest that cellular ubiquitin synthesis and recycling functions were independent of Sem1.

A possible function of Sem1 on the ubiquitination pathway was analysed. The last step of the ubiquitination cascade is the attachment of ubiquitin to target substrates by neddylated E3 ubiquitin cullin RING ligases (CRLs). CRLs are under the control of the COP9 signalosome and its deneddylase Csn5/CsnE. COP9 is required for sexual fungal development and physically interacts with Den1/DenA, a second conserved deneddylase, which promotes asexual development [14–16]. The *Δsem1* strain showed reduced amounts of neddylated (65%) and unneddylated Cul1/CulA (40%) compared to wildtype (Fig 3C). The majority of the CulA proteins were in their unneddylated and inactive form. RT-PCRs revealed that the expression levels of *csnE*, *culC* or *culD* genes were decreased in the *Δsem1* mutant strain compared to wildtype, whereas *denA* transcripts were unchanged (S2A Fig). This could be due to Sem1’s chaperon functions for the assembly of various protein complexes involved in transcription, RNA splicing or nuclear export [2].

Reduced levels of neddylated cullins and subsequently a limited ubiquitin conjugation of substrates in the absence of Sem1 suggest an important function of Sem1 in the ubiquitination pathway of target proteins due to its impact on transcription and on proteasome assembly and function.

### Sem1 increases the stability of the intrinsic lid deubiquitinating enzyme Rpn11

Accelerated proteolysis due to increased numbers of proteasomes can cause decreased levels of ubiquitin conjugates [17, 18]. Transcript levels of genes encoding proteasomal subunits or ubiquitin receptors were compared between strains with or without Sem1 to examine whether increased transcription contributes to decreased ubiquitination observed in the *Δsem1* strain (Figs 3B and S2B).

The *rpn3* transcription of wildtype cells was significantly higher than in the *Δsem1* mutant strain, where transcription was reduced. The *rpn3* gene encodes a protein which is tethered by Sem1 to the proteasome lid during biogenesis and interacts in the mature lid with Rpn7 [19]. The reduced transcription of rpn3 transcripts suggest limited incorporation of Rpn3 protein into the 26S proteasome.

The *rpn10* gene encodes one of the intrinsic ubiquitin receptors of the proteasome and is located at the interface of the regulatory particle between the base and the lid. Expression of *rpn10* as well as of other *rpn* transcripts for the lid subunits were similar in strains with or without Sem1, except for *rpn11* mRNA (Figs 3B and S2B).

The transcription level of *rpn11* encoding the intrinsic ubiquitin isopeptidase of the 26S proteasome was doubled in the mutant strain compared to wildtype. Controlled *rpn11* expression using an inducible ^*P*^*TetOn*-*rpn11* fusion gene was applied to examine whether increased amounts of *rpn11* transcripts result in higher deubiquitinase activity and reduce the overall population of ubiquitinated proteins. The ^*P*^*TetOn*-*rpn11* strain was only able to grow in the presence of a threshold concentration of at least 5μg/ml doxycycline, indicating that Rpn11 is essential for growth (S3A Fig). Eight-fold increase in the transcription of *rpn11* did not affect the expression of the control genes *sem1* or *csn5*, but led to an overall decrease in ub-conjugated proteins compared to wildtype (S3B and S3C Fig). This supports that significantly increased Rpn11 isopeptidase activity can contribute to the reduction in ubiquitin conjugates as observed in the *Δsem1* mutant strain (Fig 3A). Cellular Rpn11 protein levels for the deubiquitinating enzyme were compared to ubiquitin receptor Rpn10 levels in cells with or without Sem1. The genes encoding Rpn11 or Rpn10 were replaced by functional Rpn-GFP fusions. Rpn10-GFP derived from *Δsem1* or wildtype strain resulted in stable Rpn10 protein levels. In contrast, Rpn11-GFP was instable and resulted in only 35% of full-length protein in the *Δsem1* strain compared to wildtype (Fig 3D).

These data imply a possible effect of Sem1 on the transcription of specific proteasomal genes. Increased *rpn11* transcripts result in less full-length Rpn11 protein in a *Δsem1* mutant strain lacking the conserved zinc–binding site in the MPN+ domain (S3D Fig), suggesting that Sem1 supports cellular Rpn11 stability. Reduced amounts of an intact 26S proteasomes observed by electron microscopy correlate with the reduced Rpn11 protein levels in *Δsem1*. Incorporation of the deubiquitination enzyme into the 26S proteasomes presumably provides a Sem1-mediated Rpn11 stabilization in fungal wildtype cells.

### Sem1 is enriched in the nucleus, whereas the ubiquitin receptor Rpn10 abundance in cytoplasm and nucleus is low

**Fig 4:**
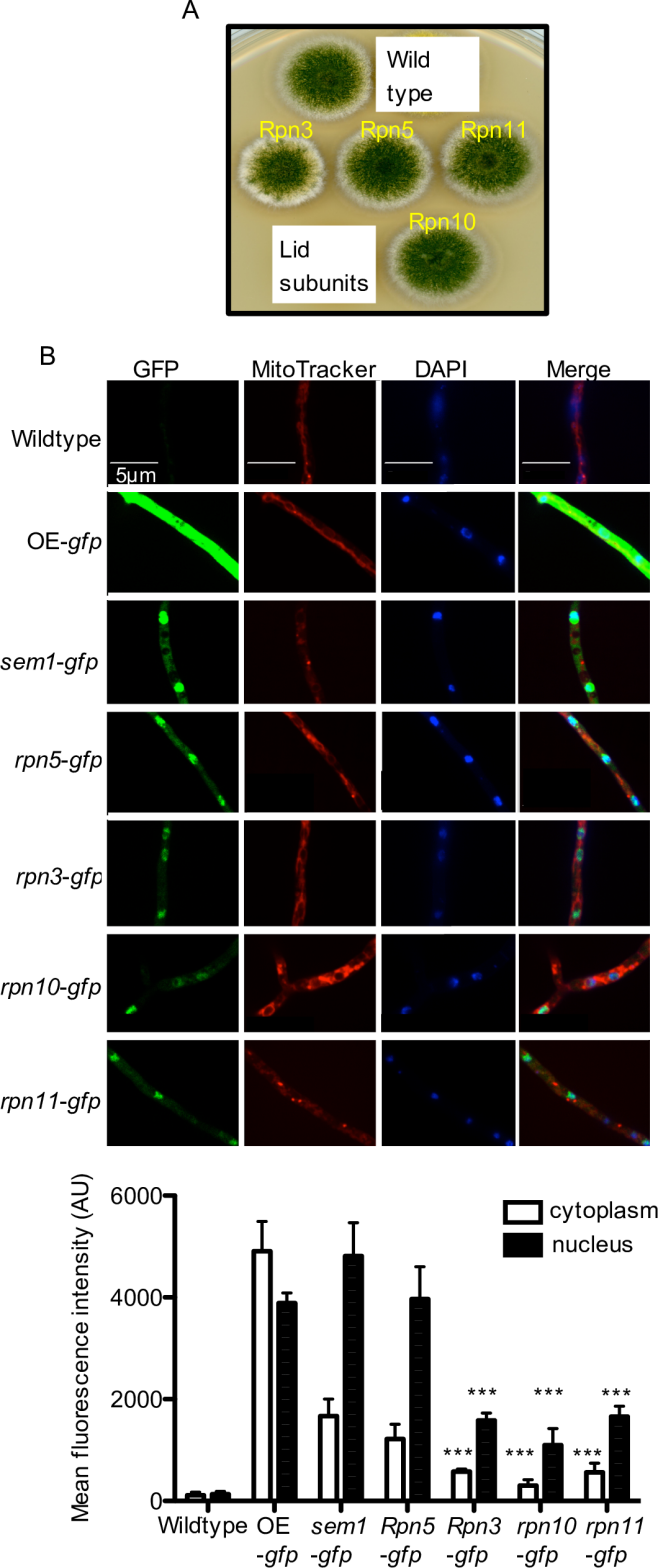
Functional GFP-tagged 19S RP subunits consist of a prominent nuclear and a smaller cytoplasmic subpopulation. **(A)** Indicated 19S RP subunits fused to GFP are functional and support asexual development. **(B)** Subcellular localization of GFP-tagged 19S RP strains determined by fluorescence microscopy. OE-overexprssion strain. The images show green GFP fluorescence (left) for 19S RP subunits, MitoTracker Red (second from left) for mitochondria, DAPI for nuclei (third from the left), and an overlay (most right). The fluorescence intensities in the cytoplasm and nucleus for the indicated GFP strains are shown as mean fluorescence intensity. Significance of differences was calculated with t-test compared to *sem1*-*gfp*, ***p<0.001.

The cellular localization of functional GFP fusions of Sem1 and the four RP subunits ubiquitin receptor Rpn10, deubiquitinating protein Rpn11, its inhibitor Rpn5, and Rpn3 that is tethered by Sem1 were compared (Fig 4A). Identical microscopy settings and the same number of spores were used for cultivation to obtain relative concentrations of 19S RP subunits in the hyphae, reflected by GFP intensities.

Significant nuclear staining was observed in the *sem1*-*gfp* strain including a minor cytoplasmic and a major nuclear Sem1 subpopulation (Fig 4B). The weakest monitored GFP signal in the cytoplasm and the nucleus was observed for Rpn3, Rpn10 and Rpn11, indicating similar cytoplasmic and nuclear abundance. Rpn5-GFP and Sem1-GFP had similar intensities, which were significantly higher than the Rpn3, Rpn10 or Rpn11 levels. Rpn5 inhibits the Rpn11 deubiquitinase and the increased intensities of Rpn5 might reflect its importance to reduce false DUB activity. Increased cellular Sem1 levels might be required, because it is not only part of the RP of the proteasome but also functions as chaperone in the assembly of several other complexes for cellular processes including transcription.

### Sem1 interacts with the TREX2 and Csn12-Thp complexes independently of the proteasomal lid

**Fig 5:**
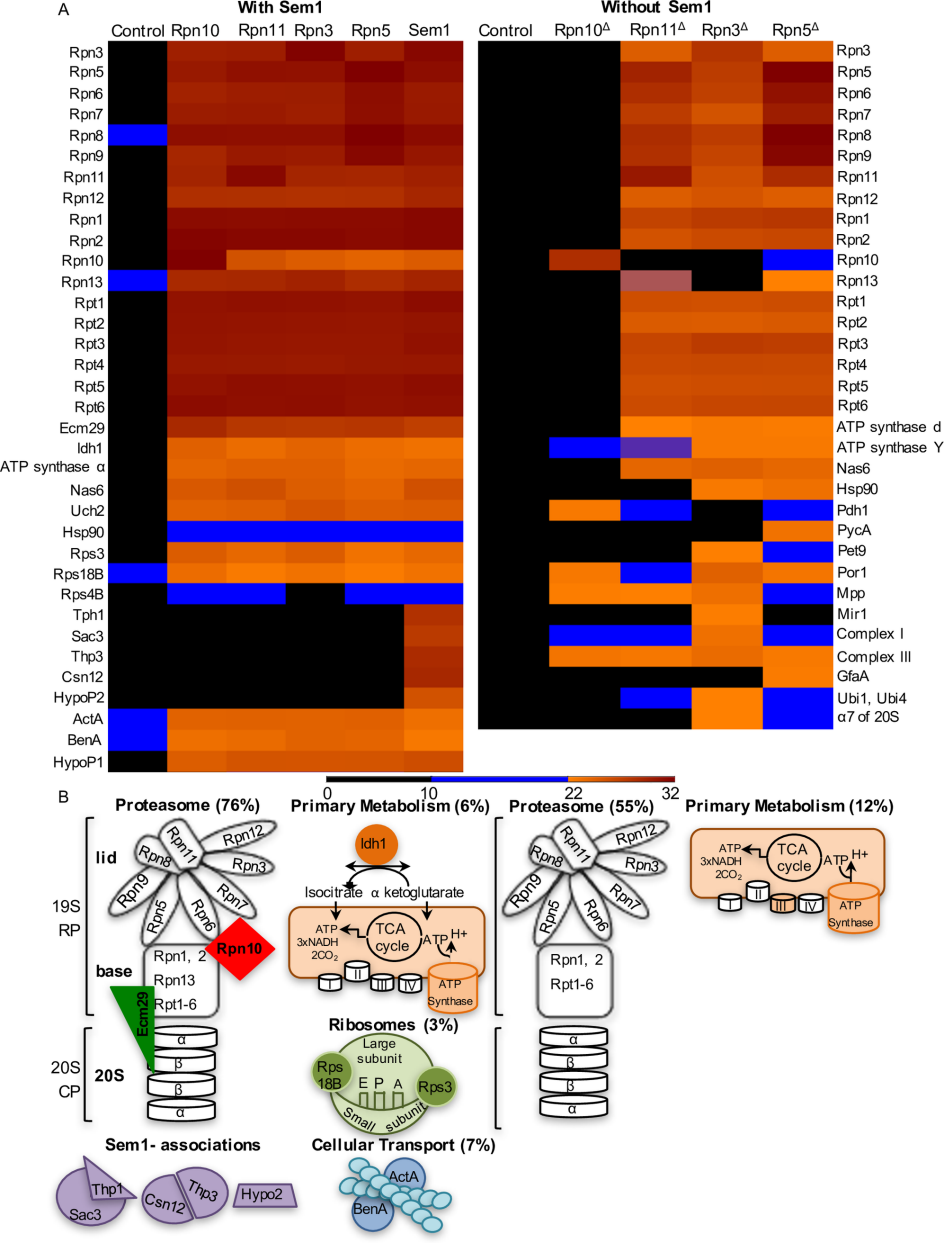
Proteins co-identified with Sem1 and 19S RP in the presence or absence of Sem1. **(A)** 34 proteins associated with 19S RP proteins fused to GFP in strains carrying Sem1. Proteins were identified in three biological replicates plotted as heat map representing LFQ intensities. HypoP1 and HypoP2 are conserved hypothetical proteins, with orthologs only among *Aspergillus*-related species. HypoP1 (AN2234) has no known conserved domain, whereas HypoP2 (AN4931) contains an Acyl-coenzyme A synthetase/AMP-(fatty) acid ligase domain (accession number COGO365). **Lower panel**: schematic representation of 30 proteins plotted in a heat map. Nas6, Hsp90, Uch24 and HypoP1 are not represented. **(B)** 33 proteins associated with 19S RP strains lacking *sem1* (marked with^Δ^) identified in two biological replicates plotted as heat map representing LFQ intensities. **Lower panel**: representation of 20 proteins identified with all RP. 9 proteins are not represented including Nas6, Hsp90, Mpp and 6 mitochondria related proteins. Proteins identified with only one tagged RP protein and/or with MSMS counts ≥5 were not represented (total 4). Heat maps representing MS/MS counts were plotted in S5 Fig. Overexpressing GFP strain served as control. In the area of the heat map were LFQ intensities and MS/MS counts are low, proteins were considered identified only when both criteria were fulfilled: LFQ>22, MS/MS counts >4. (see also S4 and S5 Figs and S1 and S2 Tables).

Affinity purifications of endogenously GFP-tagged Sem1, Rpn3, Rpn5, Rpn11 and Rpn10 combined with subsequent protein identification by mass spectrometry resulted in 34 putative interaction partners for Sem1 and 29 for Rpn GFP-fused subunits (Fig 5).

Sem1-GFP recruited two proteins of the transcription export complex 2 (TREX2) and two proteins homologous to subunits of the yeast transcription regulator complex, Csn12-Thp3, in agreement with previous approaches [2]. These associations were not observed with the other lid subunits and were Sem1-specific. An additional Sem1-specific association was found with the hypothetical protein HypoP2 (encoded by the AN4931 gene), which is conserved among 21 *Aspergillus* species but not in the unicellular yeasts *S*. *cerevisiae* or *S*. *pombe*.

29 proteins were identified associating both with Sem1 and the other GFP- tagged 19S RPs. The 29 interaction partners were grouped into five clusters: (1) protein degradation by the proteasome, (2) proteins involved in mitochondria-related activities, (3) proteins associated with ribosomes, (4) tubulin of the cytoskeleton and (5) conserved hypothetical protein HypoP1 (encoded by AN2234) with orthologs only in *Aspergillus*-related species and no conserved domain (Fig 5A lower panel). These associations point to a link between Sem1 as part of the regulatory particle and protein homeostasis, transport and mitochondria-related activities.

### Sem1 associates with the intact 19S regulatory particle and is required for the incorporation of the ubiquitin receptor Rpn10 and the recruitment of the tethering factor Ecm29

GFP pull-downs corroborated that *A*. *nidulans* Sem1 associates as part of the lid, with the complete 19S RP consisting of all 19S RP subunits (Fig 5A). Four identified *in vivo* interactions, which were also identified with the other analysed 19S RP subunits, support an important contribution of Sem1 to proteasome assembly, enabling the lid to associate with the base and the 19S RP to associate with the 20S CP. The Sem1-Rpn10 interaction might stabilize the connection between the proteasome lid and the base [20, 21]. The Sem1 interactions with base and lid associated chaperons, namely Nas6 (PSMD10 in human) and Hsp90, corroborates a Sem1 assembly function [22–25]. Sem1 also interacted with Ecm29, which stabilizes the 26S proteasome by tethering the 20S CP to the 19S RP [23].

Neither the ubiquitin receptor Rpn10 nor the tethering protein Ecm29 could be identified with any of the *rpn*-*gfp* strains when Sem1 was absent (Fig 5B). Consistently, in the absence of Sem1, Rpn10-GFP failed to pull any of the base and lid related proteasome subunits (Fig 5B). This supports an *in vivo* function of Sem1 through the ubiquitin receptor Rpn10 and Ecm29 for the interaction of base, lid and CP, which is essential for the assembly of intact 26S proteasomes.

### Sem1 interacts *in vivo* with the ubiquitin receptor Rpn10 and the intrinsic lid deubiquitinating enzyme Rpn11

**Fig 6:**
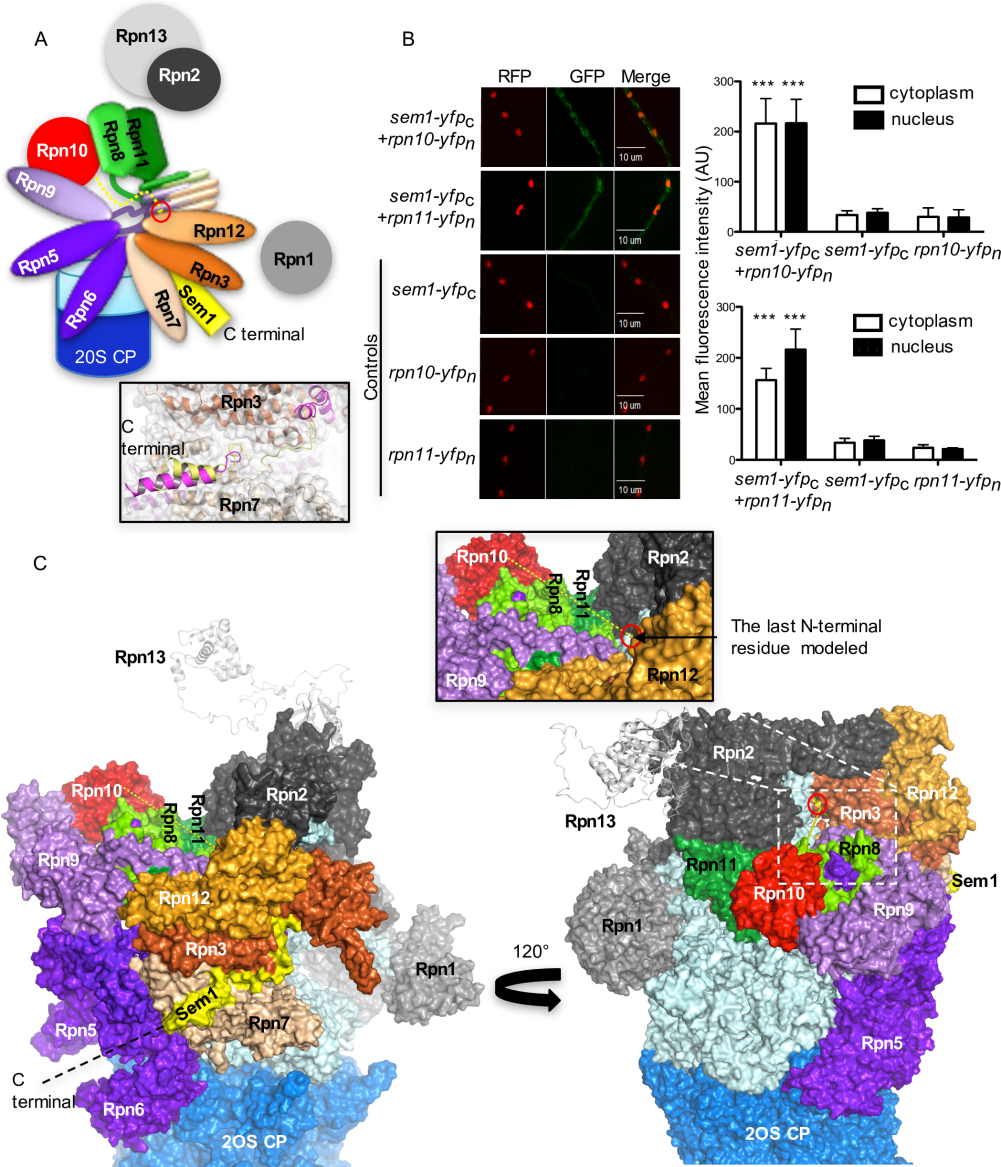
Sem1 interacts *in vivo* with Rpn10 and Rpn11. **(A)** Schematic model of the orientation of N- and C-termini of Sem1 (yellow) within the lid associated to the base (light blue) and the 20 core particle of the 26S proteasome. The red circle represents the last N-terminal amino acid residue modelled in Sem1. Zoomed in positions of the C-terminal region of human (Dss1 in magenta) and yeast (yellow) Sem1 are depicted in frame. **(B)** Bimolecular fluorescence complementation studies (BiFC) of Sem1 fusion proteins with the C-terminal half of yellow fluorescent protein co-expressed with either Rpn10 or Rpn11 fusions to N-terminal YFP result in specific signals corroborating physical interaction in fungal cells (*A*. *nidulans* strains: sem1-yfp_c_+*rpn10-yfp*_*n*_ or *sem1-yfp*_*c*_+*rpn11-yfp*_*n*_). Nuclei were visualized in red by expression of *rfp-h2A*. The fluorescence intensities both in the cytoplasm and nucleus for the indicated strains are shown as mean fluorescence intensity. Significance of differences was calculated with t-test compared to the control strains expressing either *sem1-yfp*_*c*_, *rpn10-yfp*_*n*_ or *rpn11-yfp*_*n*_, ***p<0.001, n = 10. Significantly higher fluorescence was observed in sem1-yfp_c_+*rpn10-yfp*_*n*_ or *sem1-yfp*_*c*_+*rpn11-yfp*_*n*_ compared to the control strains. **(C)** Homology model of the 26S proteasome from *A*. *nidulans* based on the human proteasome cryoEM structure (EMDB-4002, PDBs: 5L4K and 5L46). The model depicts two surface representations of the 19S RP with C-terminal region of Sem1 located at the interface formed by the N-terminal domain of Rpn3 and Rpn7 (yellow surface). The model of Sem1 is missing the N-terminally fragment of approximately 30 residues. 120° rotation depicts the last N-terminal amino acid residue modelled, located in a cleft formed by Rpn3 (red circle). It is conceivable that the missing N-terminal region of Sem1 can pass through this cleft and form interactions with Rpn10 and Rpn11 within the lid (dashed yellow line).

The domain architecture of the lid of the proteasome is conserved in eukaryotic cells (Fig 6A). The *Δsem1* mutant strain possesses a lid where the Rpn10 ubiquitin receptor is missing and the Rpn11 deubiquitinase protein levels are reduced, although *rpn11* transcript levels are increased. This suggests that Sem1 supports the assembly of stable functional capped 26S proteasomes by a molecular mechanism, which includes the physical interaction between Sem1 and Rpn10 to assemble lid to base and that Sem1 protects Rpn11 protein integrity, which is required for the specific ATP/ubiquitin-dependent 26S proteasome activity.

Bimolecular fluorescence complementation studies (BiFC) were performed to determine whether Sem1 interaction with Rpn10 and Rpn11 can be monitored in fungal cells *in vivo*. Fungal strains expressing functional Sem1 fused through its C-terminus to the C-terminal half of YFP and C-terminal Rpn10 and Rpn11 fusions to the N-terminal half of YFP were examined (strains *sem1-yfp*_c_*+rpn10-yfp*_n_ and *sem1-yfp*_c_*+rpn11-yfp*_n_, respectively). The fluorescence observed in strains containing fused Sem1-Rpn10 and fused Sem1-Rpn11 was significantly higher compared to the respective control strains (Fig 6B right panel). These cellular interactions of Sem1 could be due to an escorting of these proteins for lid assembly.

A homology model of the *A*. *nidulans* 19RP based on the cryoEM structure of human proteasome was generated (EMDB-4002, PDBs: 5L4K and 5L46 [26]) to examine the possibility of interactions between Sem1-Rpn10 and/or Sem1-Rpn11 upon assembly of the lid (Fig 6C). The modeled C-terminal fragment of Sem1 is bound in a structurally conserved cleft between the lid subunits Rpn3 and Rpn7. This structural conservation results in a very similar binding mode of Sem1 observed for yeast and human proteasomes (Fig 6A). In that binding mode, the extension of Sem1 towards the N-terminus reaches to the other side of the lid (opposite) due to an opening in the center of the lid. The missing (not modeled) N-terminal tail, comprising approximately 30 amino acids, could be responsible for direct interactions of Sem1 with both Rpn10 and Rpn11 forming the opposite surface of the lid (Fig 6C).

These data corroborate direct interactions between Sem1-Rpn10 as well as Sem1-Rpn11 in the fungal cell. Sem1 might escort Rpn10 and Rpn11 proteins to the lid and support the assembly and positioning of both proteins into a stable capped 26S proteasome through the assistance of its flexible N-terminal tail.

### Lid subunits from *sem1*-deficient strain associate with mitochondria related proteins

**Fig 7:**
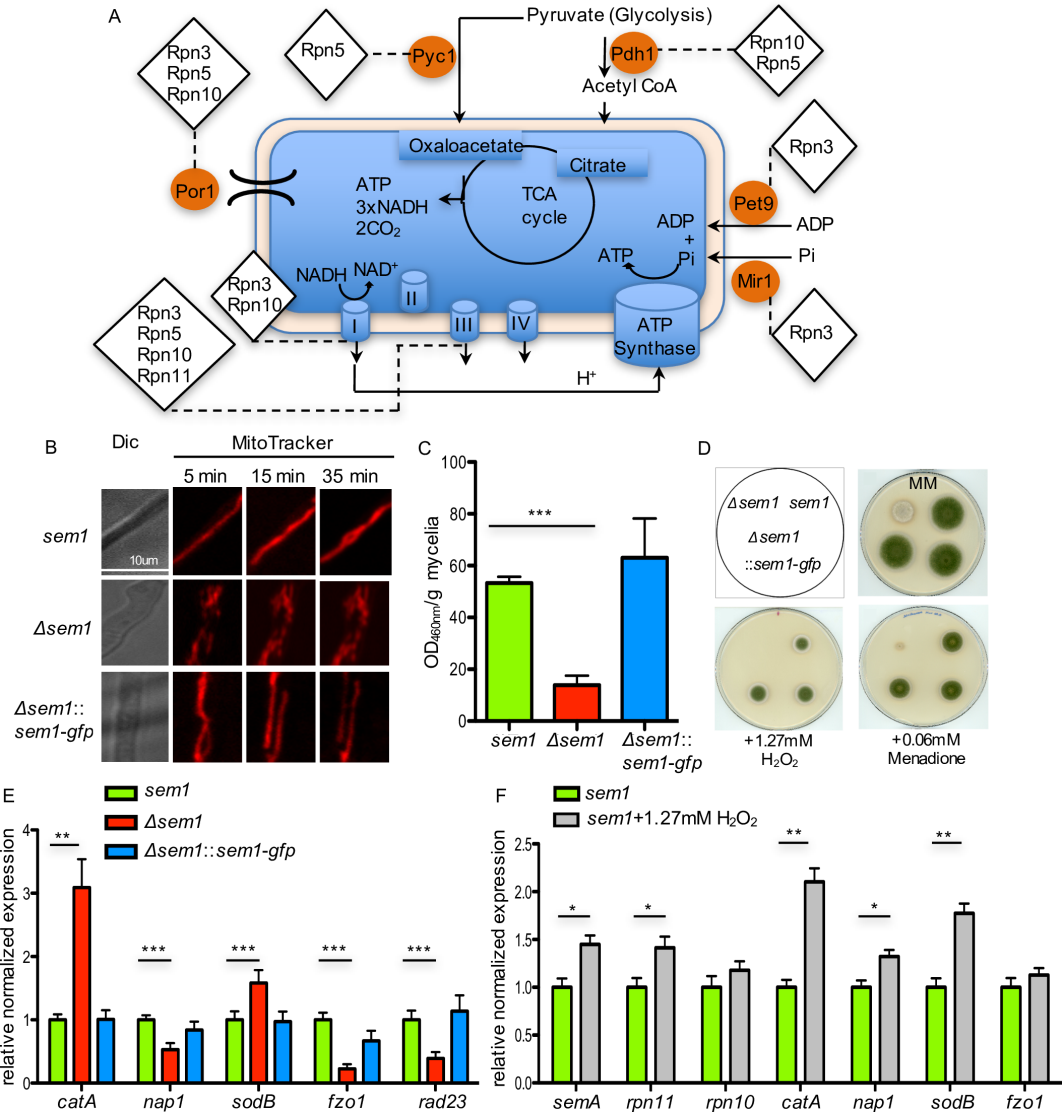
Sem1 is required for an appropriate oxidative stress response in *A*. *nidulans*. **(A)** 19S RP without Sem1 associates with TCA cycle and respiratory chain related proteins. Interacting enzymes are marked with orange circles and the respective 19S regulatory particle subunits associated with them are indicated with diamonds. **(B)** Mitochondria morphology is compromised in *Δsem1* mutant strain as observed by time-lapse microscopy with the fluorescence marker MitoTracker. Hypha from wildtype, complementation and deletion strain were observed 5 min, 15 min and 35 min after the addition of the MitoTracker. Scale bar: 10 μm. **(C)** The total cellular NADH production is reduced in the *Δsem1* mutant strain. n = 5, ***p<0.001. **(D)** Oxidative stress inducing compounds inhibit *Δsem1* colony growth. Respective strains were spotted on minimal medium plates (MM, control) and MM plates supplemented as indicated, n = 4. Two complementation strains served as internal biological replications. **(E)** *Δsem1* mutant strain induced the transcription of the antioxidants encoding genes *catA* and *sodB*. Results are shown as relative expression compared to *sem1*, n = 3, ***p<0.001. **(F)** *sem1* and *rpn11* are induced as response to oxidative stress in *sem1*+1.27mM H_2_O_2_. Bars represent mean values of four independent experiments, ***p<0.001 (see also S6 Fig).

In contrast to wildtype, the 19S regulatory particle lacking Sem1 associated to proteins related to NADH or ATP production (Figs 5 and 7A). The dihydrolipoamide acetyltransferase Pdh1 (AN6708) is part of the pyruvate dehydrogenase complex for the oxidative decarboxylation of pyruvate to acetyl-CoA. Cytoplasmic Pcy1 (AN4462) converts pyruvate to oxaloacetate. Both enzyme products are used by the mitochondrial TCA cycle. The Sem1-interacting protein Rpn3 associated only in the absence of Sem1 with the ADP/ATP carrier Pet9 (AN4064) of the mitochondrial inner membrane, which exchanges cytosolic ADP for mitochondrial synthesized ATP. Rpn3, Rpn5 or Rpn10 interacted in the absence of Sem1 with the mitochondrial porin Por1 (AN4402). This outer membrane protein is required for maintenance of mitochondrial osmotic stability and membrane permeability. The 19S RP without Sem1 associates with the β-subunit of the mitochondrial processing protease Mpp (AN0747), which cleaves the N-terminal targeting sequence from mitochondrial-imported proteins. Subunit II of complex III (AN8373) and NADH-ubiquinone oxidoreductase, complex I (AN4288) are two components of the mitochondrial inner membrane electron transport chain which interact with RPs without Sem1. These findings suggest a specific physical interaction of RP subunits in the absence of Sem1 with mitochondria, which is not found when Sem1 is present.

### *A*. *nidulans* requires Sem1 for cellular NADH production and the mitochondria integrity

The morphology of the mitochondria in *Δsem1* and wildtype strains were compared to examine the impact of the association of lid subunits with the TCA cycle and respiratory chain related proteins, which were exclusively found in the absence of Sem1 (Fig 7A). The mitochondria of *Δsem1* cells showed dots of disrupted filaments and differed significantly from the wildtype (Fig 7B). This phenotype suggests a defect in the dynamic equilibrium between mitochondria fusion and fission processes, that could be caused by the physical interactions of *Δsem1* RPs with the mitochondrial machinery, which is suppressed in wildtype where Sem1 is present.

The total cellular NADH production was determined in *Δsem1* and compared to wildtype (Fig 7C). Strains expressing Sem1 or Sem1-GFP showed similar high concentrations of NADH produced per gram mycelium after 20h of vegetative growth (63.0±15.2 and 53.3±2.4 ΔOD_460nm_/g mycelium, respectively). Deleting *sem1* resulted in only 26% of NADH compared to wildtype (13.84±3.7 ΔOD_460nm_/g mycelium). The fragmented mitochondria observed in the mutant strain might be defective and less active compared to mitochondria in the wildtype strain.

### Sem1 is required for an appropriate fungal oxidative stress response

A *Δsem1* mutant strain showed fragmented mitochondria, produced less NADH and accumulated orange/red pigments (Fig 7B–7D). Mutant strains with a deficient COP9 signalosome or CAND-proteins controlling cellular cullin E3 ubiquitin ligase activities also accumulated red dyes and were linked to a misregulated secondary metabolism and an inappropriate oxidative stress response [27, 28]. Consistently, *Δsem1* mutant strain was not able to grow on hydrogen peroxide and could hardly grow in the presence of menadione, whereas strains with functional Sem1 germinated and produced normal looking colonies (Fig 7D). The oxidative stress response was monitored at the transcriptional level of three superoxide dismutase encoding genes *sodA*, *sodB* and *sodM* and the catalase encoding gene *catA* (Figs 7E and S2B). Deletion of *sem1* resulted in significant 2.5-fold up-regulation of transcripts for catalase A and 1.6-fold increase for superoxide dismutase B compared to wildtype (Figs 7E and S2B). These results underline a critical Sem1 function in the oxidative stress response. The mutant strain presumably tries to minimize the damaging effects of ROS caused by the damaged mitochondria, thereby inducing an antioxidative defence system.

### Oxidative stress induces increased *sem1* and proteasomal deubiquitinase *rpn11* transcript levels

Wildtype mitochondria were not damaged when exposed to moderate oxidative stress. Transcription levels of mitochondrial genes *fzo1* for fusion or *fis1* and *dnm1* for fission were similar in fungal wildtype cells with an intact Sem1 in absence or presence of oxidative stress. The encountered oxidative stress was reflected in a response of increased expression of catalase encoding *catA*, *sodB* for a superoxide dismustase or the regulatory gene for oxidative stress *nap1* corresponding to yeast *yap1* (Figs 7F and S6).

It was analysed, whether Sem1 expression levels varied in response to oxidative stress. The inflicted oxidative stress resulted in a significantly increased transcription of *sem1* and *rpn11* genes (Fig 7F). This increasing level of *sem1* and *rpn11* transcripts represents a yet undescribed physiological cellular oxidative stress response, which might protect the cell from increased 20S proteasome levels. Increased transcription to produce more Sem1 and Rpn11 proteins might counteract the damaging interaction of aberrant 19S regulatory particles with the mitochondria, which is detrimental for vegetative cells and for fungal differentiation, requiring an oxidative stress signal as developmental trigger [29, 30].

## Discussion

**Fig 8:**
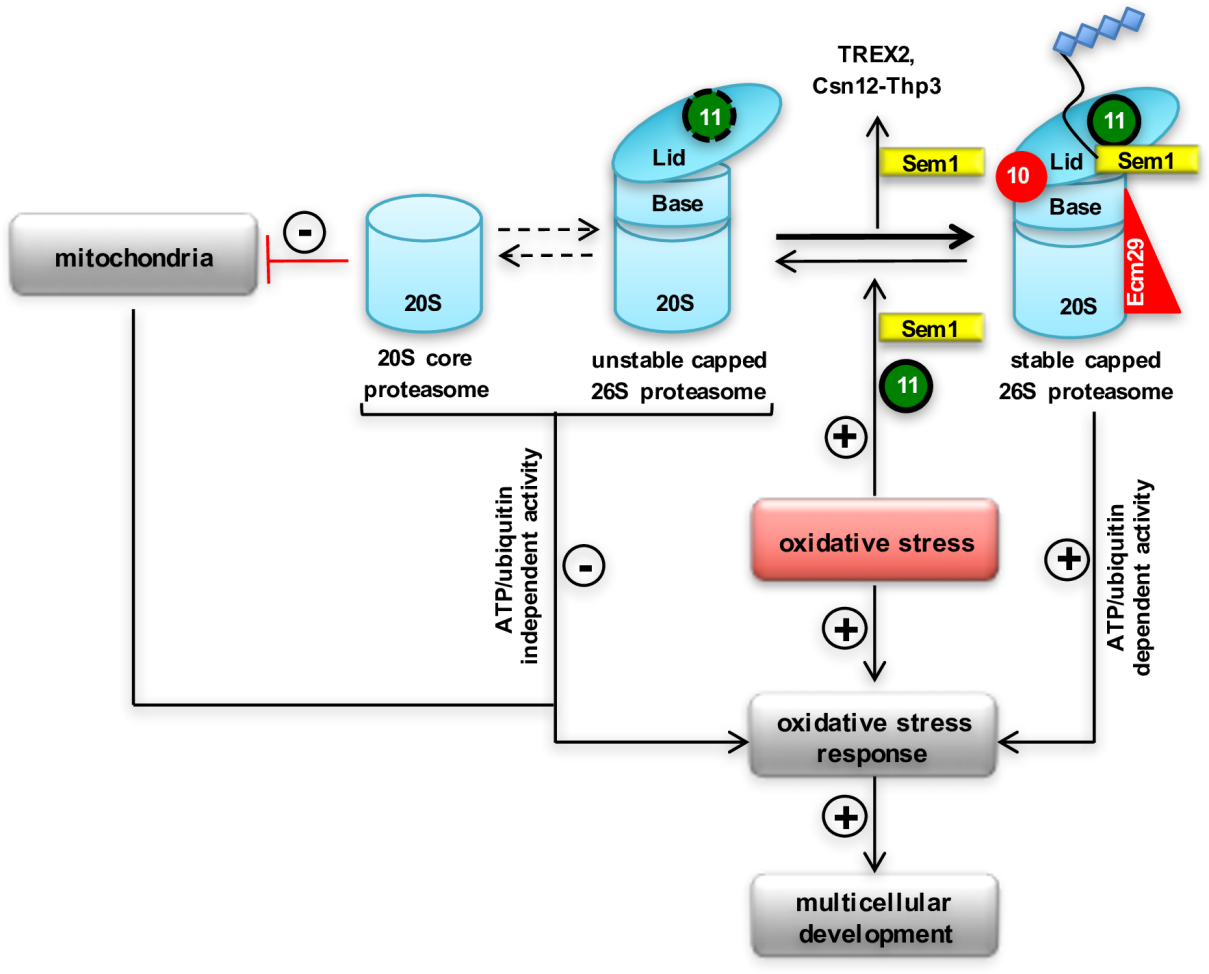
Molecular Sem1 function and *A*. *nidulans* multicellular development. Sem1 links cellular redox state, mitochondria integrity, efficient assembly of several complexes including the 26S proteasome to multicellular differentiation. Oxidative stress induces the oxidative stress response. Multicellular development requires an internal reactive oxygen species (ROS) as a signal as well as protection against oxidative stress. Oxidative stress induced transcription of genes for proteasome subunits *sem1* and *rpn11* (indicated with +). Capped proteasomes, which can be formed in the presence or absence of Sem1, differ in composition and quantity. Sufficient amounts of Sem1 and Rpn11 are required during oxidative stress to provide correctly assembled 26S proteasomes including the ubiquitin receptor Rpn10, the tethering factor Ecm29 and stabilized full-length deubiquitinating enzyme Rpn11. Consequently, these stable capped 26S proteasomes are fully functional and can selectively catalyse ubiquitinated proteins (indicated as diamonds) in an ATP/ubiquitin-dependent manner. Decreased amounts of Sem1 result in damaged mitochondria, unstable capped 26S proteasomes and increased levels of activated oxidation-driven 20S core proteasomes, which can efficiently hydrolyse proteins in an ATP/ubiquitin-independent manner.

The conserved Sem1 protein supports the assembly of multiple cellular complexes and represents the ninth *bona fide* subunit of the 19S regulatory particle of the 26S proteasome. A novel cellular function was detected, which connects the proteasome function and the cellular redox state at the molecular level. Oxidative imbalances in the multicellular ascomycete *Aspergillus nidulans* resulted not only in increased transcription of genes for detoxification enzymes such as catalases, but also in increased transcription of *sem1* and *rpn11* encoding the proteasomal deubiquitinating enzyme. Sufficient amounts of Sem1 and Rpn11 proteins are necessary during oxidative stress to provide higher amounts of correctly assembled 26S proteasomes. A lack of Sem1 resulted in increased oxidation-driven 20S proteasomes and instable capped proteasomes lacking the Rpn10 ubiquitin receptor, a functional Rpn11 deubiquitinating enzyme and the chaperone Ecm29 that connects the CP to the RP. Decreased amounts of Sem1 compromise multicellular fungal development, which requires internal reactive oxygen signals as triggers (Fig 8).

Sem1 is required for morphological integrity and functionality of the mitochondria, evident by the structural defects caused by the absence of Sem1. A physiological link between dysfunctional mitochondria due to mistransferred proteins and a proteostatic response had been described [31]. Lack of Sem1 results in a five-fold decrease in cellular NADH production compared to a wildtype strain. Consistently, Sem1 is required to allow fungal vegetative growth in the presence of oxidative-stress inducing compounds such as H_2_O_2_ or menadione. An appropriate oxidative stress response therefore includes in a wildtype fungus not only increased transcription of genes for detoxificating enzymes such as *catA* or *sodB* genes, but also increased transcripts for subunits of the 19S RP of the proteasome such as *sem1* or *rpn11*. This corroborates that increased protein levels for these subunits are part of the cellular answer to stress. Mutant strains without Sem1 protein are hypersensitive towards stress, although they constitutively induce transcription of genes for detoxification enzymes. The Sem1-dependent stress response is linked to coordinated fungal secondary metabolism. This link is reminiscent to genetic studies with mutant strains defective in COP9 signalosome or CAND proteins, which control the activity of cullin E3 ubiquitin ligases. Impaired function of COP9 signalosome, CAND or Sem1 results in a redox imbalance and in accumulation of red orcinol derived secondary metabolites visible in the fungal colony as red dye [27, 28].

Multicellular development specifically requires protection against oxidative stress, as internal cellular stress signals are required for the progression of differentiation. This includes transient increase in reactive oxygen species (ROS) for developmental programmes in animals [32] or fungi [29, 30]. In fungi, increased ROS production interferes with hyphal fusion as one of the initial steps from vegetative hyphal growth to multicellular development [33]. In humans, increased ROS production is associated with mitochondria disorders, aging or neurodegenerative diseases, where unfolding of oxidized proteins promotes accumulation of protein aggregates [34, 35].

Sem1 of the unicellular yeast had been proposed to stabilize the interactions between the lid and the base [36]. In multicellular *A*. *nidulans*, Sem1 is not only required for correct 26S assembly, but represents a lid subunit which is mandatory for 26S proteasome composition, stability and specificity [36–38]. *A*. *nidulans* can form a lid without Sem1, as it had been described for *S*. *cerevisiae*, where a comparison between wildtype and *Δsem1* lids by single particle cryo-EM analyses revealed significant structural differences with rearrangements of Rpn3 and Rpn7 in the *Δsem1* lid [5, 39]. Sem1-dependent functions on assembly and stabilization of *A*. *nidulans* 26S proteasome were visualized by negative staining electron microscopy, where *Δsem1*-deficient proteasomes from mutant strains consist mostly of 20S proteasomes with only low abundance of 26S proteasome complexes. Sem1 fulfils its stabilization function by the recruitment of Rpn10, which is mandatory to stabilize the interaction between lid and base [19, 21]. In addition to the incorporation of Rpn10, Sem1 is required for the recruitment of Ecm29 as facilitator, which associates the 19S regulatory particle to the 20S core particle. Direct interactions between Sem1 and the base were not reported, but Rpn10 makes extensive connections with different lid and base subunits, namely the Rpn11/Rpn8 heterodimer, Rpn9 and Rpn12 or the base subunits Rpn1 and the Rpt4/Rpt5 heterodimer [40–43]. *A*. *nidulans* Rpn10 is a stable protein, which is unable to associate with any lid or base subunit without Sem1. The molecular function of Sem1 could be to escort Rpn10 to the lid and to stabilize it during the assembly. The homology model of the 19S regulatory particle of *A*. *nidulans* positions the C- terminal fragment of Sem1 between Rpn3 and Rpn7 and the last modeled N-terminal residue of Sem1 in the cleft formed by Rpn3. This cleft is located in a close proximity to an opening in the centre of the lid, which could be a structural feature allowing the N-terminal tail of Sem1 to pass through and interact with Rpn10 and Rpn11. Thereby, it is conceivable that this cleft accommodates the N-terminal tail of Sem1 and stabilizes the interaction of Rpn10 and Rpn11 within the lid. This is in agreement with recent cross-linking experiments between the N-terminal part of Sem1 and C-terminal part of Rpn11 in the fully assembled lid but not in LP2 (lid particle 2), the lid intermediated consisting all eight lid subunits except of Rpn12 [44]. A cross-linking between Sem1 and Rpn10 was not yet described. Modelling the corresponding regions of Sem1 from human or *S*. *cerevisiae* into the *A*. *nidulans* model leads to similar results. The *in vivo* BiFC study show direct physical interaction between Sem1 and Rpn10 and supports an escorting and assembly function of Sem1 for Rpn10 prior and during 26S proteasome assembly. Conclusively, the data suggests that the presence of Sem1 is a prerequisite to Rpn10 and is essential for accurate and efficient assembly of a stable capped 26S proteasome.

Oxidation drives 26S disassociation presumably by posttranslational modifications of α5, α6, α7 rings of the 20S CP [45, 46]. It was demonstrated that S-glutathionylation through redox-regulation promotes gate opening of the 20S CP, which is otherwise closed unless 19S RP is bound to it [47]. Cellular proteolysis of the oxidation-driven 20S proteasomes derived from the *Δsem1* mutant strain impaired in redox regulation resulted in higher degradation rates compared to the wildytype 20S proteasomes. As ATP/ubiquitin-independent degradation requires 20S proteasome complexes but no ATP and no polyubiquitinated proteins [48], this presumably allows the *Δsem1* strain to maintain efficient degradation. In the mutant strain the 20S proteasomes are kinetically favoured regardless of the absence of 19S RP or ATP. The *Δsem1* mutant strain produces less NADH, which potentially reduces oxidative respiration and ATP production.

Mutant analysis revealed that the presence of the sem1 gene correlated with increased ubiquitin-conjugates and reduced ATP/ubiquitin-independent degradation in comparison to the mutant strain lacking sem1. A ubiquitin receptor function has been described for the human counterpart Dss1 with a ubiquitin binding site overlapping the Rpn3-Rpn7 binding sites [49]. This suggests that Sem1 can associate with the proteasome leaving the two acidic patches available for ubiquitinated substrates and/or can even dynamically associate with the proteasome to escort ubiquitinated substrates to the proximity of proteasomes [2]. The observed decreased levels of ubiquitinated substrates in the *A*. *nidulans* mutant strain lacking *sem1* could be related to CRLs and the attachment of ubiquitin to substrates. In yeast, the deletion of Sem1 resulted in an accumulation of ubiquitinated substrates presumably due to non-functional proteasomes [3]. Decreased cellular levels of ubiquitin-conjugates were also observed in mammalian epithelial cells exposed to H_2_O_2,_ as a consequence of the oxidation of cysteine residues in the active sites of E1-E3 ubiquitin-conjugating enzymes [50–52]. In *A*. *nidulans*, oxidative stress not only reduces the amount of the E3 Cul1 scaffold subunit, but also influenced the transcription of *culC* for Cul3, *culD* for Cul4 and *csnE* for the COP9 signalosome subunit Csn5. This suggests that ubiquitinated proteins are regulated in *A*. *nidulans* in response to oxidative stress where ATP/ubiquitin-independent degradation takes place.

Sem1 represents a novel molecular link between proteasome assembly and specificity, mitochondrial integrity and cellular development. The viable *Δsem1* mutant strain from *A*. *nidulans* is a valuable tool to investigate ATP/ubiquitin-independent proteolysis to elucidate the cross talk between cullins, COP9 signalosome and proteasome in response to oxidative stress. Understanding Sem1 function during mitochondrial stress will provide new insights for our understanding of mitochondrial-associated pathogenesis. Increased Sem1 activity might delay mitochondria dysfunction and can be used for further therapies. Elucidating the mechanisms by which Sem1 affects and regulates oxidative stress is beneficial in the efforts to understand and treat age-related human diseases and explore potential therapies.

## Materials and methods

### Ethics statement

The study did not involve human participants, specimens or tissue samples, or vertebrate animals, embryos or tissues.

### Strains, media and cultivation

*A*. *nidulans* strains used in this study are listed in Table A (S1 File-supporting information). The *A*. *nidulans sem1* gene corresponds to *semA* in the fungal nomenclature. Spore concentration was determined by Z2 Coulter particle count and size analyser (Beckmann counter). Vegetative growth was performed in flasks with indentations containing supplemented liquid media and 5x10^5^ spores/ml at 37°C for 20 hours. For asexual and sexual development 10 μl of 10000 spores/ml were spotted on supplemented MM and incubated at 37°C. For the time point experiment, asexual spores were counted with Thoma chamber. Incubating the plates in constant white light triggered asexual sporulation, whereas sexual fruiting body formation was induced by oxygen limitation (the plates were sealed) and darkness. Asexual spores were counted using Thoma chamber. For sexual growth, 100 μl of 1x10^6^ spores/ml were spared on supplemented MM and incubated at 37°C and incubated in the dark with limited oxygen for 7 and 10 days.

### Negative staining EM

Samples were bound to a glow discharged carbon foil covered grid. After staining with 1% uranyl acetate, the samples were evaluated with a CM 120 transmission electron microscope (FEI, Eindhoven, and The Netherlands). Images were taken with a TemCam F416 CMOS camera (TVIPS, Gauting, Germany).

### NADH quantification

The total NADH production was measured as described earlier [53]. 8x 5ml mycelia of each strain were used for the measurements. Activity was measured at 460nm and was normalized to DCW (g mycelium) after drying for 3 days at 60°C.

### Proteasome purification and activity

Proteasomes were purified from 8 g grained mycelium using rapid 26S proteasome purification kit from UBPBio. Concentrations were determined by Nanodrop and activity assays were performed according to manufacturer recommendations. The activity was measured in an assay buffer containing 12.5 mM Tris HCl pH = 7.5+10 mM KCl+1.25 mM MgCl_2_+0.125 mM ATP+0.25 mM DTT+0.0125 mg/ml BSA. The release of AMC from 100 μM fluorgenic peptide, Suc-LLVY-AMC, was measured over 30–60 min and background fluorescence was subtracted from all measurements.

### Statistical analysis and quantification

t-test was used to determine significance of the results (http://www.quantitativeskills.com/sisa/statistics/oneway.htm). The intensity of western blot bands was determined with ImageJ v1.48 analysis software. Mean intensities from biological replicates (n) were relative to glyceraldehyde-3-phosphate dehydrogenase (GAPDH) serving as loading control and were normalized to wildtype (%). Expression levels assayed by RT-PCR are shown as relative expression compared to wilidtype and represents mean value and standard error of the indicated independent experiments (n).

## Supporting information

S1 Fig Fungal Sem1 resembles its human counterpart. Sequence alignments of Sem1 protein from *A*. *nidulans* (An), *S*. *pombe* (Sp, 52%/48%), humans (Hs, 47%/66%), and *S*. *cerevisiae* (Sc, 50%/69%). UniProt accession numbers: AN1245, O14140, P60896 and O94742, respectively. The secondary structures of Sem1 from *A*. *nidulans* indicated (orange cylinder, α-helix) were predicted by the Psi-blast-based secondary structure prediction (PSIPRED; http://bioinf.cs.ucl.ac.uk/psipred/). Percentage represent sequence identity/sequence similarity, respectively. Sequences were aligned using ClustalW2 with asterisk (*) indicating fully conserved residues, colon (:) conservation between groups of strongly similar properties and period (.) conservation between groups of weakly similar properties (related to Fig 1).
(TIF)

S2 Fig Transcript levels of cullins, deneddylases, superoxide dismutases (SOD) and 19S RP in *Δsem1* mutant strain. **(A)** Reduced transcript levels of *culC*, *culD* and *csn5/csnE* in *Δsem1* mutant strain. Results are shown as relative expression compared to *sem1*. The plot represents the mean value and standard error of the mean of four experiments. T-test of *Δsem1* vs. *sem1*, ****p<0.0001. **(B)** Unchanged transcript levels of genes for antioxidants and selected genes for 19S RP subunits in *Δsem1* mutant strain are shown. Results are shown as relative expression compared to *sem1*. The plot represents the mean value and standard error of the mean of five experiments. All transcript levels were determined after 20h of vegetative growth (related to Fig 3).
(TIF)

S3 Fig The effect of Rpn11 protein on the overall population of ubiquitinated proteins and the stability of Rpn11 in *Δsem1* mutant strain. **(A-C)** Inducing the transcription of *rpn11* resulted in decreased levels of ub-conjugated proteins compared to WT (wildtype, *sem1*). **(A)** Doxycycline-dependent growth of ^*P*^*TetOn*-*rpn11*. 10,000 spores were spotted on MM supplemented with the indicated concentrations of doxycycline. Plates were incubated at 37°C for 3 days. **(B)** Transcript levels of *rpn11*, *sem1* and *csn5* in the presence of 20μg/ml doxycycline. *sem1* (0μg/ml Doxy, green), *sem1* (20μg/ml Doxy, black) and ^*P*^*TetOn*-*rpn11* (20μg/ml Doxy, gray). Strains were grown vegetatively at 37°C for 20h prior to the extraction of total RNA. The expression was assayed by quantitative RT-PCR. Results are shown as relative expression compared to *sem1* without doxycycline (green). The plots represent the mean value and standard error of the mean of at least five independent experiments. T-test of *Δsem1* vs. *sem1*, p<0.01. **(C)** Decrease in polyubiquitinated substrates was observed upon induction of *rpn11* strain. Proteins were extracted after 20h of vegetative growth at 37°C from strains grown in the presence of 5μg/ml and 20μg/ml doxycycline. 40μg total proteins were loaded in each lane. Polyubiquitinated substrates were detected with α-ubiquitin and glyceraldehyde-3-phosphate dehydrogenase (GAPDH) served as loading control. The ubiquitin/GAPDH intensities from two biological replicates were quantified by ImageJ v1.48 and normalized to the respective *sem1* (%). **(D)** Rpn11-GFP from *Δsem1* strain lacks the conserved zinc-binding site in MPN+ domain. MPN+ domain and the conserved zinc-binding site (in red) were identified using NCBI conserved domain database https://www.ncbi.nlm.nih.gov/Structure/cdd/wrpsb.cgi (related to Fig 3).
(TIF)

S4 Fig 19S RP subunits fused to GFP are functional. **(A)** Expression leveles of lid subunits fused to GFP in *A*. *nidulans*. The respective strains were grown vegetatively at 37°C for 20h prior to the extraction of proteins. 40μg total proteins were loaded in each lane. GFP tagged lid subunits were detected with α-GFP and glyceraldehyde-3-phosphate dehydrogenase (GAPDH) served as loading control. No GFP signal was detected in the negative control (*sem1*, wildtype), whereas the positive control (OE-GFP) showed a prominent band at the expected size of free GFP. The expected MW of the tagged proteins represent: 28.38, 99.31, 84.83, 57.94, 66.17 and 38.63 KDa, for free GFP, Rpn3-GFP, Rpn5-GFP, Rpn10-GFP, Rpn11-GFP and Sem1-GFP, respectively. **(B)** No change in total ubiquitin-conjugated substrates was observed in the GFP tagged lid subunits. Proteins were extracted after 20h of vegetative growth. Ubiqutin conjugates were detected with α-Ub and GAPDH was used as loading control. The intensity of the respective bands was determined with ImageJ v1.48 analysis software. Mean intensities from two biological replicates were normalized to the loading control GAPDH. **(C)** Asexual growth of tagged lid subunits in the absence of *sem1*. Equal numbers of spores (10,000 spores) of the respective strains were spotted on MM and grown at 37°C for 3 days in light. Top view is presented. The *sem1* strain (wildtype) showed normal asexual growth while the GFP-tagged strains lacking *sem1* showed reduced growth and accumulation of a reddish pigment, which is reminiscent to a fungal strain defective in the COP9 signalosome. **(D)** Ubiqutin-conjugates decrease in 19S RP strains lacking *sem1*. Proteins were extracted after 20h of vegetative growth. Ubiqutin conjugate proteins were detected with α-Ub (related to Fig 5).
(TIF)

S5 Fig MS/MS counts of proteins associated with Sem1 and 19S RP in the presence or absence of *sem1*. **(A)** Identified proteins were plotted according to MS/MS counts. Proteins were classified as identified, if the total number of unique peptides identified was ≥3, and the protein was present in at least two out of three biological repeats. Numbers represent the respective proteins identified in each group. Heat maps were generated by MaxQuant and plotted using Perseus. For precise log2 intensity and MS/MS counts of all proteins in this figure refer to S1 & S2 Tables (related to Fig 5). Proteins in an area of low MSMS count were considered identified only if both criteria were fulfilled: LFQ>22, MS/MS counts >4. **Left panel-** 41 proteins associated with 19S *rpn*-*gfp* strains. Proteins were identified in three biological replicates plotted as heat map representing MSMS counts. **Right panel-** 33 proteins associated with 19S *rpn-gfp*::*Δsem1* strains. Each column represents the proteins identified in two biological replicates. **(B)** Rpn3, Rpn5 and Rpn10-GFP interact with proteins involved in TCA cycle, glycolysis and gene expression. Diamonds represent the indicated lid subunits; interactions are marked with doted lines. Pfk: phosphofructokinase (EC 2.7.1.11); Met6: methionine synthase (EC 2.1.1.13). Except for PFK, MetH and Ub, all other indicated enzymes were identified with SequestHT and Mascot. Note that except of Ubi1 and Ubi4, the indicated interactions were not observed when using 19S *rpn-gfp*::*Δsem1* strains (Fig 5), indicating that Sem1 mediates these associations (related to Fig 5).
(TIF)

S6 Fig Transcript levels of superoxide dismutases (SOD), 19S RP and cullins in *sem1* exposed to 2h of oxidative stress. Relative expression levels after 20h of vegetative growth followed by 2h of oxidative stress (1.27mM H_2_O_2_). Bars represent mean value of four independent experiments. T-test of *sem1* with H_2_O_2_ vs. *sem1*, *p<0.005 (related to Fig 7).
(TIF)

S1 File Supplementary data. (DOCX)

S1 Table List of proteins associated with 19S *rpn-gfp* strains, used for generating Figs 5 and S5. (XLSX)

S2 Table List of proteins associated with 19S *rpn-gfp*::*Δsem1* strains, used for generating Figs 5 and S5. (XLSX)
