# Supplementary figures and images for: Sem1 links proteasome stability and specificity to multicellular development
Source: PLoS Genet. 2018 Feb 5;14(2):e1007141. doi: 10.1371/journal.pgen.1007141 (PMC5821377; doi:10.1371/journal.pgen.1007141)

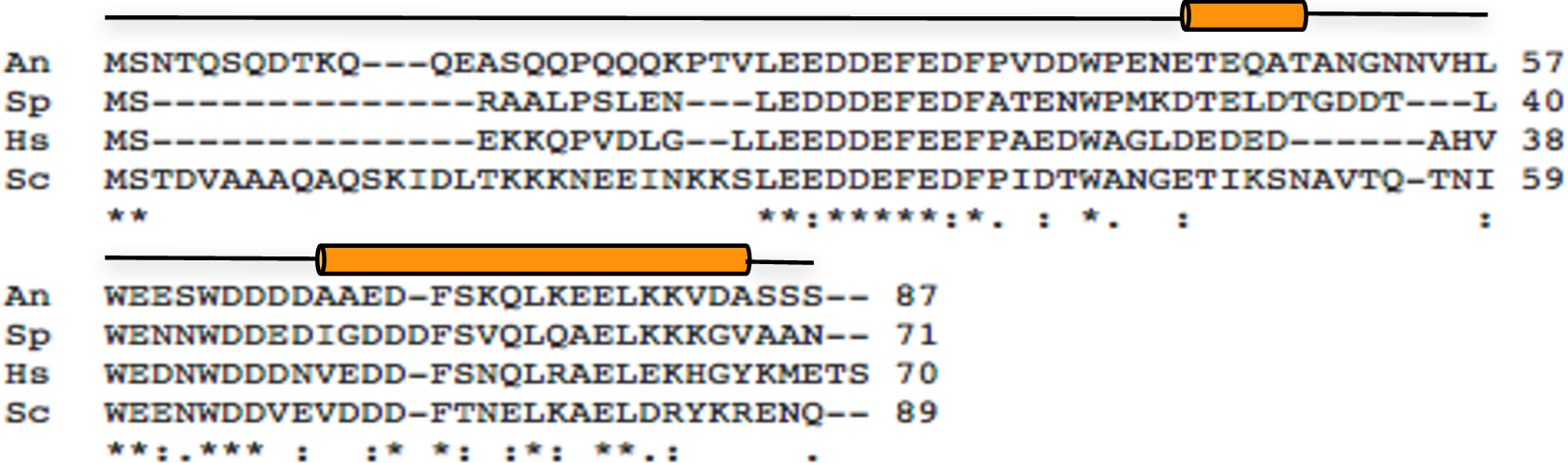

Supplement: S1 Fig — Sequence alignments of Sem1 protein from A. nidulans (An), S. pombe (Sp, 52%/48%), humans (Hs, 47%/66%), and S. cerevisiae (Sc, 50%/69%). UniProt accession numbers: AN1245, O14140, P60896 and O94742, respectively. The secondary structures of Sem1 from A. nidulans indicated (orange cylinder, α-helix) were predicted by the Psi-blast-based secondary structure prediction (PSIPRED; http://bioinf.cs.ucl.ac.uk/psipred/). Percentage represent sequence identity/sequence similarity, respectively. Sequences were aligned using ClustalW2 with asterisk (*) indicating fully conserved residues, colon (:) conservation between groups of strongly similar properties and period (.) conservation between groups of weakly similar properties (related to Fig 1). (TIF) [file pgen.1007141.s001.tif]

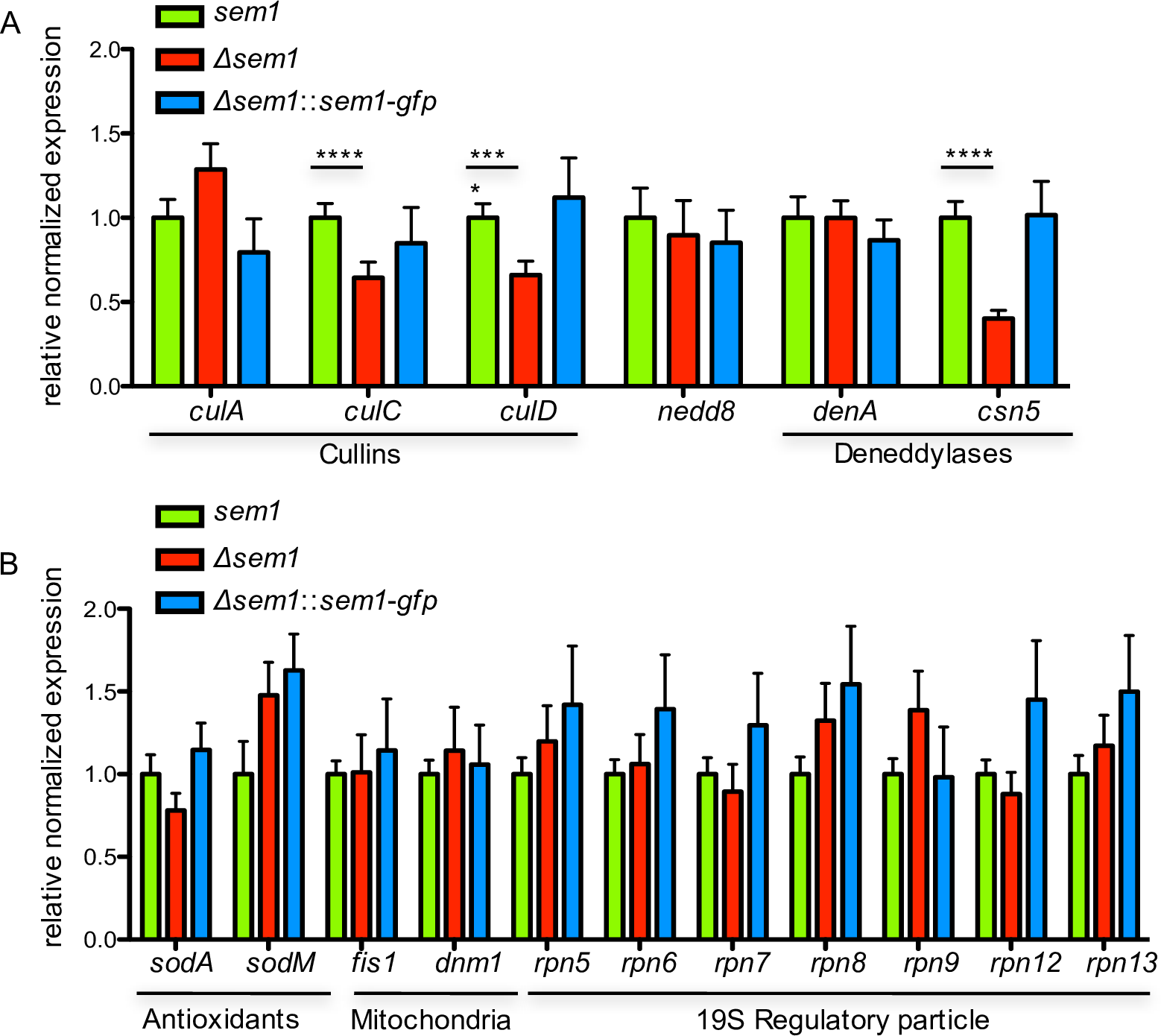

Supplement: S2 Fig — (A) Reduced transcript levels of culC, culD and csn5/csnE in Δsem1 mutant strain. Results are shown as relative expression compared to sem1. The plot represents the mean value and standard error of the mean of four experiments. T-test of Δsem1 vs. sem1, ****p<0.0001. (B) Unchanged transcript levels of genes for antioxidants and selected genes for 19S RP subunits in Δsem1 mutant strain are shown. Results are shown as relative expression compared to sem1. The plot represents the mean value and standard error of the mean of five experiments. All transcript levels were determined after 20h of vegetative growth (related to Fig 3). (TIF) [file pgen.1007141.s002.tif]

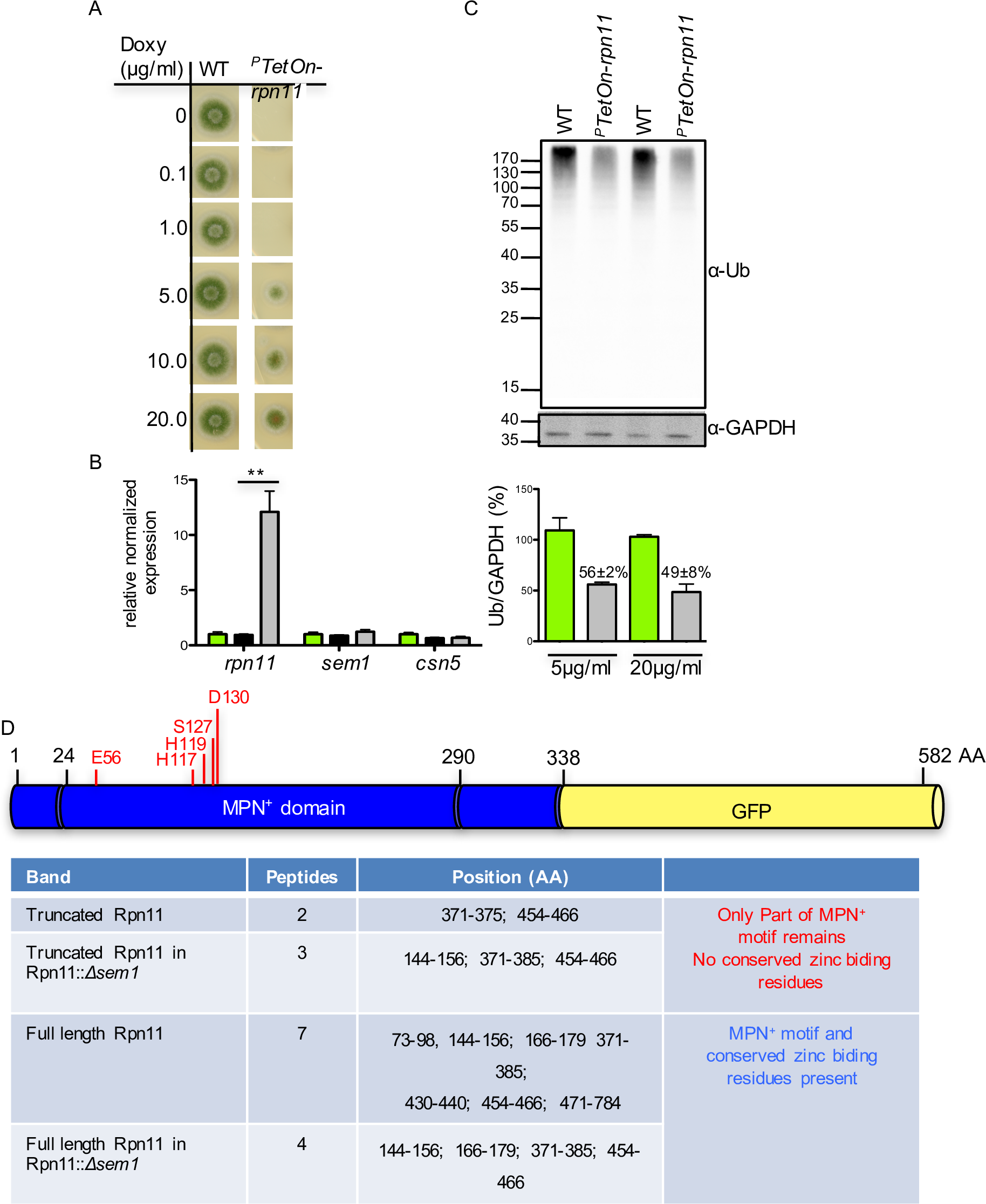

Supplement: S3 Fig — (A-C) Inducing the transcription of rpn11 resulted in decreased levels of ub-conjugated proteins compared to WT (wildtype, sem1). (A) Doxycycline-dependent growth of PTetOn-rpn11. 10,000 spores were spotted on MM supplemented with the indicated concentrations of doxycycline. Plates were incubated at 37°C for 3 days. (B) Transcript levels of rpn11, sem1 and csn5 in the presence of 20μg/ml doxycycline. sem1 (0μg/ml Doxy, green), sem1 (20μg/ml Doxy, black) and PTetOn-rpn11 (20μg/ml Doxy, gray). Strains were grown vegetatively at 37°C for 20h prior to the extraction of total RNA. The expression was assayed by quantitative RT-PCR. Results are shown as relative expression compared to sem1 without doxycycline (green). The plots represent the mean value and standard error of the mean of at least five independent experiments. T-test of Δsem1 vs. sem1, p<0.01. (C) Decrease in polyubiquitinated substrates was observed upon induction of rpn11 strain. Proteins were extracted after 20h of vegetative growth at 37°C from strains grown in the presence of 5μg/ml and 20μg/ml doxycycline. 40μg total proteins were loaded in each lane. Polyubiquitinated substrates were detected with α-ubiquitin and glyceraldehyde-3-phosphate dehydrogenase (GAPDH) served as loading control. The ubiquitin/GAPDH intensities from two biological replicates were quantified by ImageJ v1.48 and normalized to the respective sem1 (%). (D) Rpn11-GFP from Δsem1 strain lacks the conserved zinc-binding site in MPN+ domain. MPN+ domain and the conserved zinc-binding site (in red) were identified using NCBI conserved domain database https://www.ncbi.nlm.nih.gov/Structure/cdd/wrpsb.cgi (related to Fig 3). (TIF) [file pgen.1007141.s003.tif]

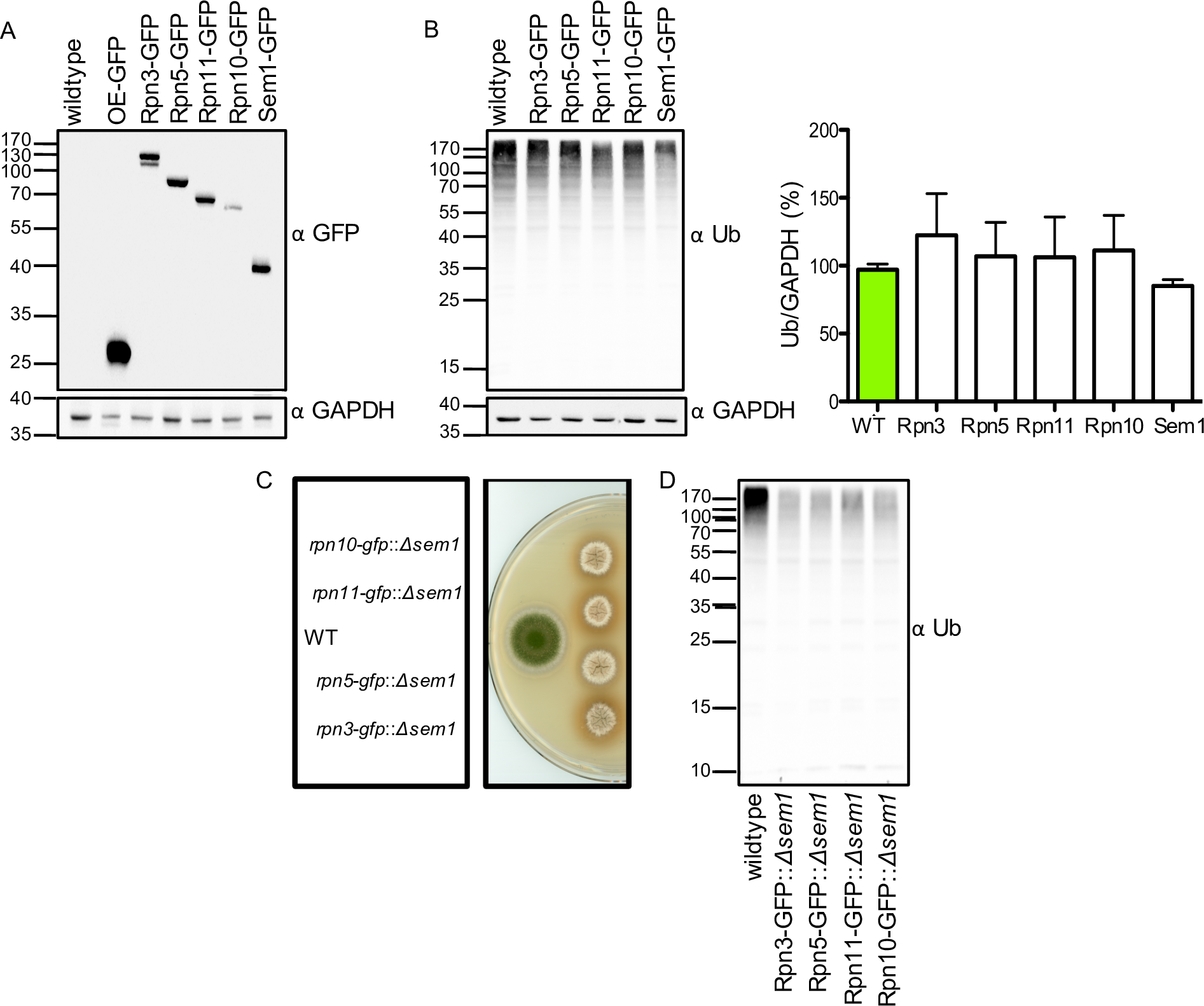

Supplement: S4 Fig — (A) Expression leveles of lid subunits fused to GFP in A. nidulans. The respective strains were grown vegetatively at 37°C for 20h prior to the extraction of proteins. 40μg total proteins were loaded in each lane. GFP tagged lid subunits were detected with α-GFP and glyceraldehyde-3-phosphate dehydrogenase (GAPDH) served as loading control. No GFP signal was detected in the negative control (sem1, wildtype), whereas the positive control (OE-GFP) showed a prominent band at the expected size of free GFP. The expected MW of the tagged proteins represent: 28.38, 99.31, 84.83, 57.94, 66.17 and 38.63 KDa, for free GFP, Rpn3-GFP, Rpn5-GFP, Rpn10-GFP, Rpn11-GFP and Sem1-GFP, respectively. (B) No change in total ubiquitin-conjugated substrates was observed in the GFP tagged lid subunits. Proteins were extracted after 20h of vegetative growth. Ubiqutin conjugates were detected with α-Ub and GAPDH was used as loading control. The intensity of the respective bands was determined with ImageJ v1.48 analysis software. Mean intensities from two biological replicates were normalized to the loading control GAPDH. (C) Asexual growth of tagged lid subunits in the absence of sem1. Equal numbers of spores (10,000 spores) of the respective strains were spotted on MM and grown at 37°C for 3 days in light. Top view is presented. The sem1 strain (wildtype) showed normal asexual growth while the GFP-tagged strains lacking sem1 showed reduced growth and accumulation of a reddish pigment, which is reminiscent to a fungal strain defective in the COP9 signalosome. (D) Ubiqutin-conjugates decrease in 19S RP strains lacking sem1. Proteins were extracted after 20h of vegetative growth. Ubiqutin conjugate proteins were detected with α-Ub (related to Fig 5). (TIF) [file pgen.1007141.s004.tif]

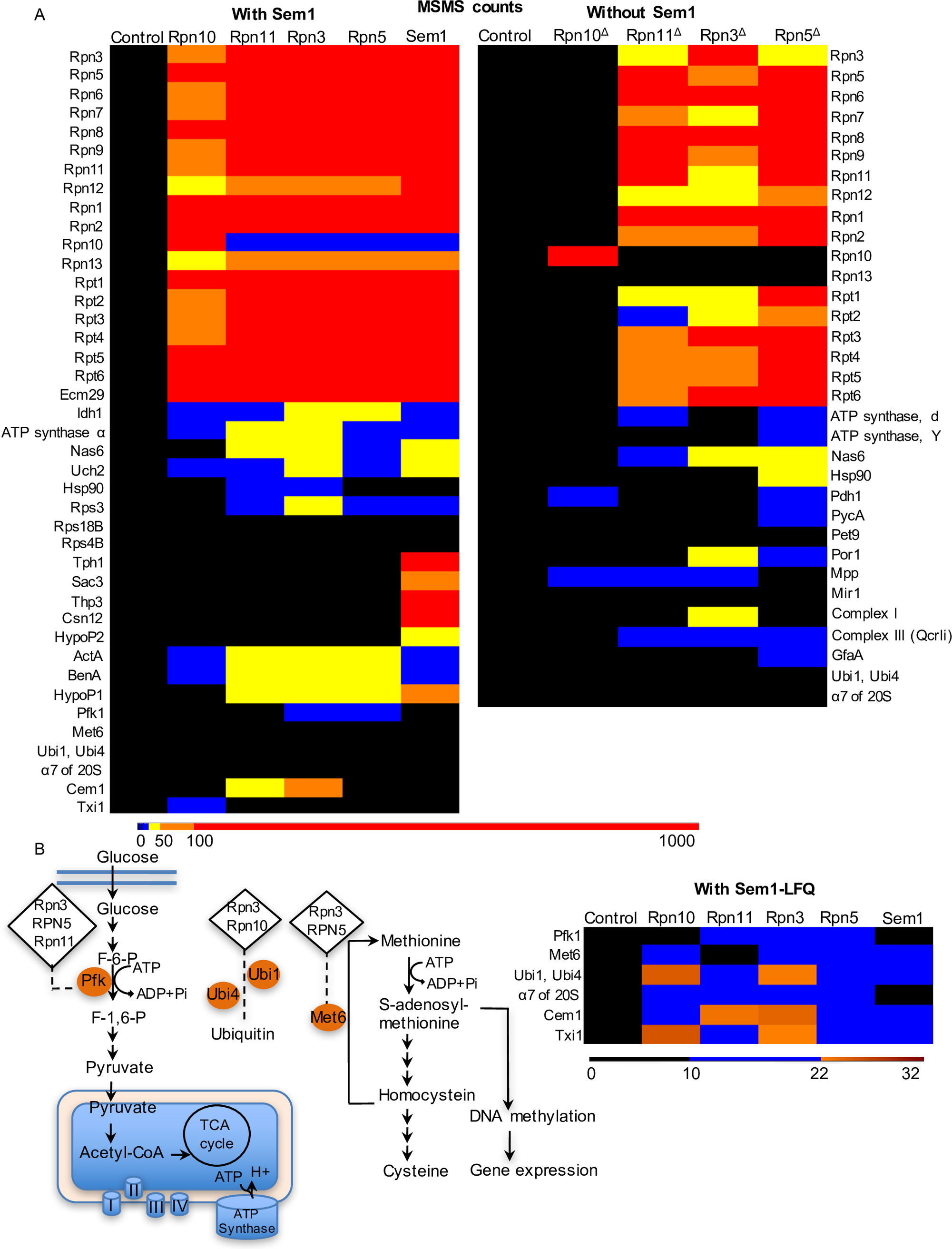

Supplement: S5 Fig — (A) Identified proteins were plotted according to MS/MS counts. Proteins were classified as identified, if the total number of unique peptides identified was ≥3, and the protein was present in at least two out of three biological repeats. Numbers represent the respective proteins identified in each group. Heat maps were generated by MaxQuant and plotted using Perseus. For precise log2 intensity and MS/MS counts of all proteins in this figure refer to S1 & S2 Tables (related to Fig 5). Proteins in an area of low MSMS count were considered identified only if both criteria were fulfilled: LFQ>22, MS/MS counts >4. Left panel- 41 proteins associated with 19S rpn-gfp strains. Proteins were identified in three biological replicates plotted as heat map representing MSMS counts. Right panel- 33 proteins associated with 19S rpn-gfp::Δsem1 strains. Each column represents the proteins identified in two biological replicates. (B) Rpn3, Rpn5 and Rpn10-GFP interact with proteins involved in TCA cycle, glycolysis and gene expression. Diamonds represent the indicated lid subunits; interactions are marked with doted lines. Pfk: phosphofructokinase (EC 2.7.1.11); Met6: methionine synthase (EC 2.1.1.13). Except for PFK, MetH and Ub, all other indicated enzymes were identified with SequestHT and Mascot. Note that except of Ubi1 and Ubi4, the indicated interactions were not observed when using 19S rpn-gfp::Δsem1 strains (Fig 5), indicating that Sem1 mediates these associations (related to Fig 5). (TIF) [file pgen.1007141.s005.tif]

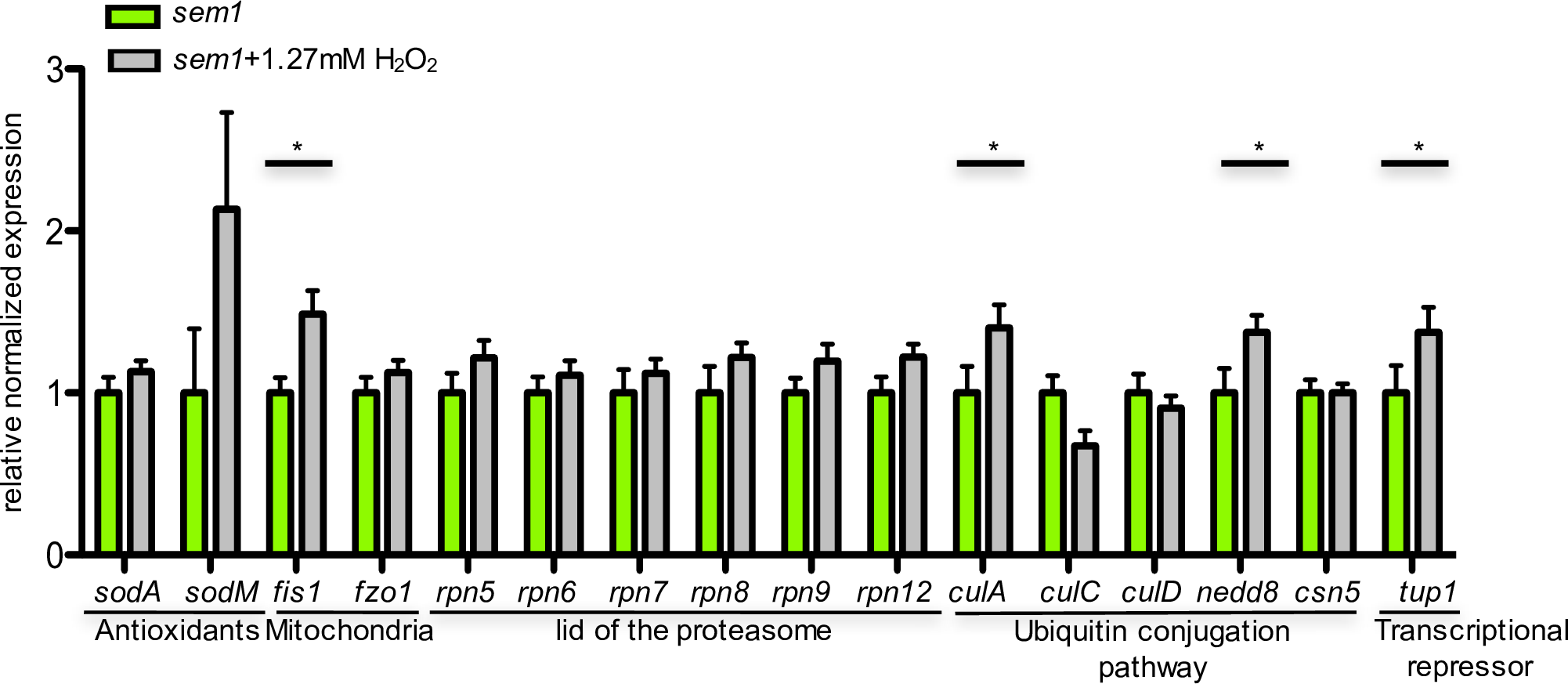

Supplement: S6 Fig — Relative expression levels after 20h of vegetative growth followed by 2h of oxidative stress (1.27mM H2O2). Bars represent mean value of four independent experiments. T-test of sem1 with H2O2 vs. sem1, *p<0.005 (related to Fig 7). (TIF) [file pgen.1007141.s006.tif]
