# Supplementary material for: Sem1 links proteasome stability and specificity to multicellular development
Source: PLoS Genet. 2018 Feb 5;14(2):e1007141. doi: 10.1371/journal.pgen.1007141 (PMC5821377; doi:10.1371/journal.pgen.1007141)
Supplement: S1 File — (DOCX) [file pgen.1007141.s007.docx]

**S1 File: Supplementary data**

**Sem1 links proteasome stability and specificity to multicellular development**

Kolog Gulko M, Heinrich G, Gross C, Popova B, Valerius O, Neumann P, Ficner R, Braus GH

**[1] Supplementary Figures pages 2-8**

**[2] Supplementary Tables pages 9-15**

**[3] Supplementary Experimental Procedures pages 16-22**

**[4] Supplementary References pages 23-24**

**[1] Supplementary Figures**

**
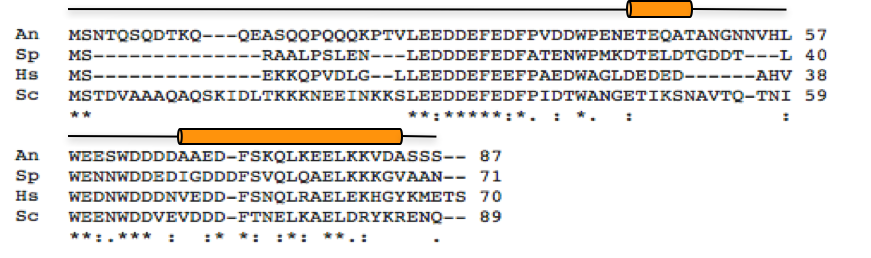
**

**S1 Fig. Fungal Sem1 resembles its human counterpart.**

Sequence alignments of Sem1 protein from *A*. *nidulans* (An), *S*. *pombe* (Sp, 52%/48%), humans (Hs, 47%/66%), and *S*. *cerevisiae* (Sc, 50%/69%). UniProt accession numbers: AN1245, O14140, P60896 and O94742, respectively. The secondary structures of Sem1 from *A. nidulans* indicated (orange cylinder, α-helix) were predicted by the Psi-blast-based secondary structure prediction (PSIPRED; http://bioinf.cs.ucl.ac.uk/psipred/). Percentage represent sequence identity/sequence similarity, respectively. Sequences were aligned using ClustalW2 with asterisk (*) indicating fully conserved residues, colon (:) conservation between groups of strongly similar properties and period (.) conservation between groups of weakly similar properties (related to Fig 1).


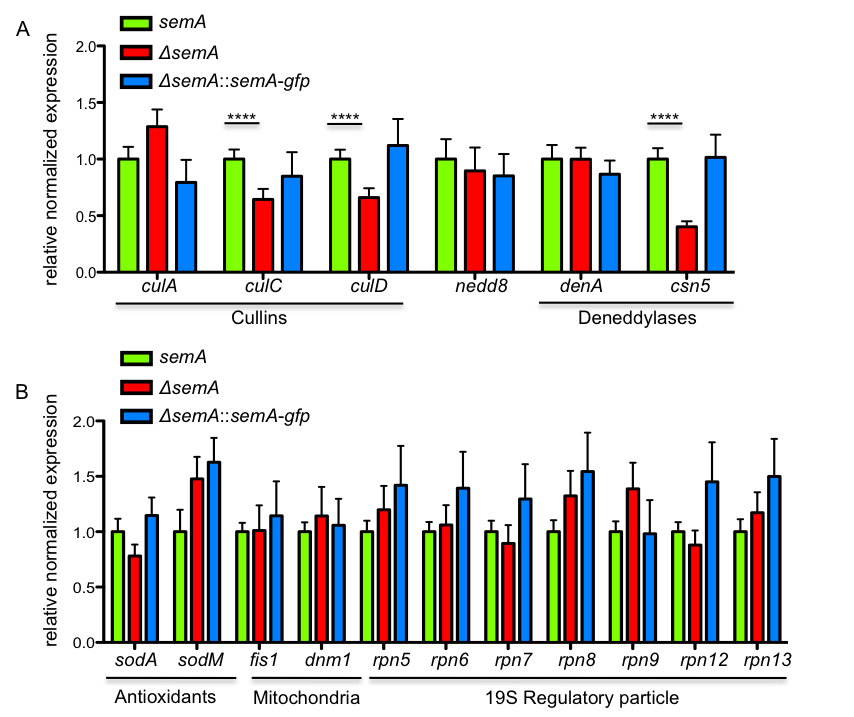


**S2 Fig. Transcript levels of cullins, deneddylases, superoxide dismutases (SOD) and 19S RP in *Δsem1* mutant strain.**

**A-** Reduced transcript levels of *culC*, *culD* and *csn5/csnE* in *Δsem1* mutant strain. Results are shown as relative expression compared to *sem1*. The plot represents the mean value and standard error of the mean of four experiments. T-test of *Δsem1* vs. *sem1*, ****p<0.0001. **B-** Unchanged transcript levels of genes for antioxidants and selected genes for 19S RP subunits in *Δsem1* mutant strain are shown. Results are shown as relative expression compared to *sem1*. The plot represents the mean value and standard error of the mean of five experiments. All transcript levels were determined after 20h of vegetative growth (related to Fig 3).


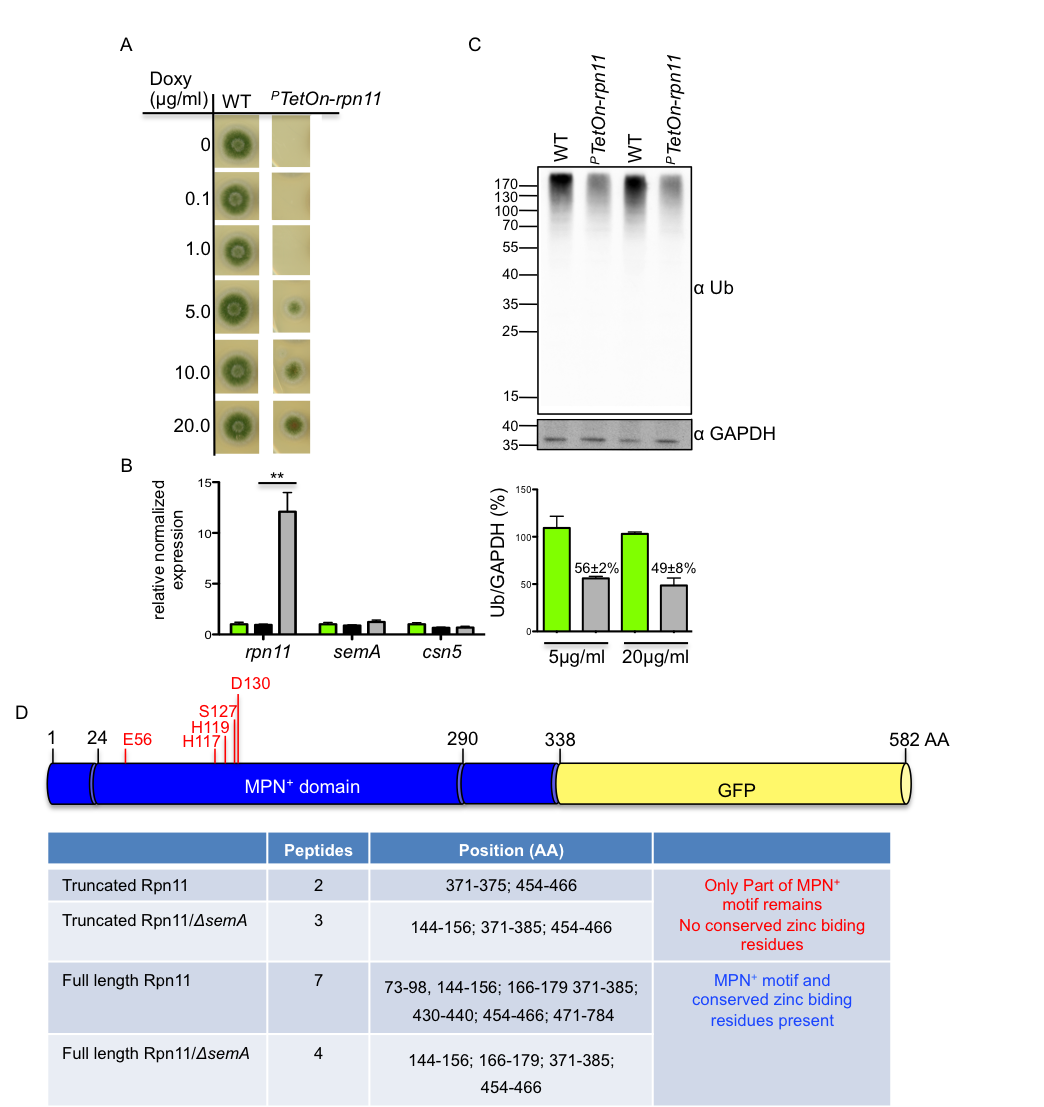


**S3 Fig.** **The effect of Rpn11 protein on the overall population of ubiquitinated proteins and the stability of Rpn11 in *Δsem1* mutant strain.**

**A-C** Inducing the transcription of *rpn11* resulted in decreased levels of ub-conjugated proteins compared to WT (wildtype, *sem1*). **A**- Doxycycline-dependent growth of *^P^TetOn*-*rpn11*. 10,000 spores were spotted on MM supplemented with the indicated concentrations of doxycycline. Plates were incubated at 37°C for 3 days. **B**- Transcript levels of *rpn11*, *sem1* and *csn5* in the presence of 20μg/ml doxycycline. *sem1* (0μg/ml Doxy, green), *sem1* (20μg/ml Doxy, black) and *^P^TetOn*-*rpn11* (20μg/ml Doxy, gray). Strains were grown vegetatively at 37°C for 20h prior to the extraction of total RNA. The expression was assayed by quantitative RT-PCR. Results are shown as relative expression compared to *sem1* without doxycycline (green). The plots represent the mean value and standard error of the mean of at least five independent experiments. T-test of *Δsem1* vs. *sem1*, p<0.01. **C-** Decrease in polyubiquitinated substrates was observed upon induction of *rpn11* strain. Proteins were extracted after 20h of vegetative growth at 37°C from strains grown in the presence of 5μg/ml and 20μg/ml doxycycline. 40μg total proteins were loaded in each lane. Polyubiquitinated substrates were detected with α-ubiquitin and glyceraldehyde-3-phosphate dehydrogenase (GAPDH) served as loading control. The ubiquitin/GAPDH intensities from two biological replicates were quantified by ImageJ v1.48 and normalized to the respective *sem1* (%). **D-** Rpn11-GFP from *Δsem1* strain lacks the conserved zinc-binding site in MPN+ domain. MPN+ domain and the conserved zinc-binding site (in red) were identified using NCBI conserved domain database <http://www.ncbi.nlm>.nih.gov/Structure/cdd/Wrpsb.cgi), (related to Fig 3).


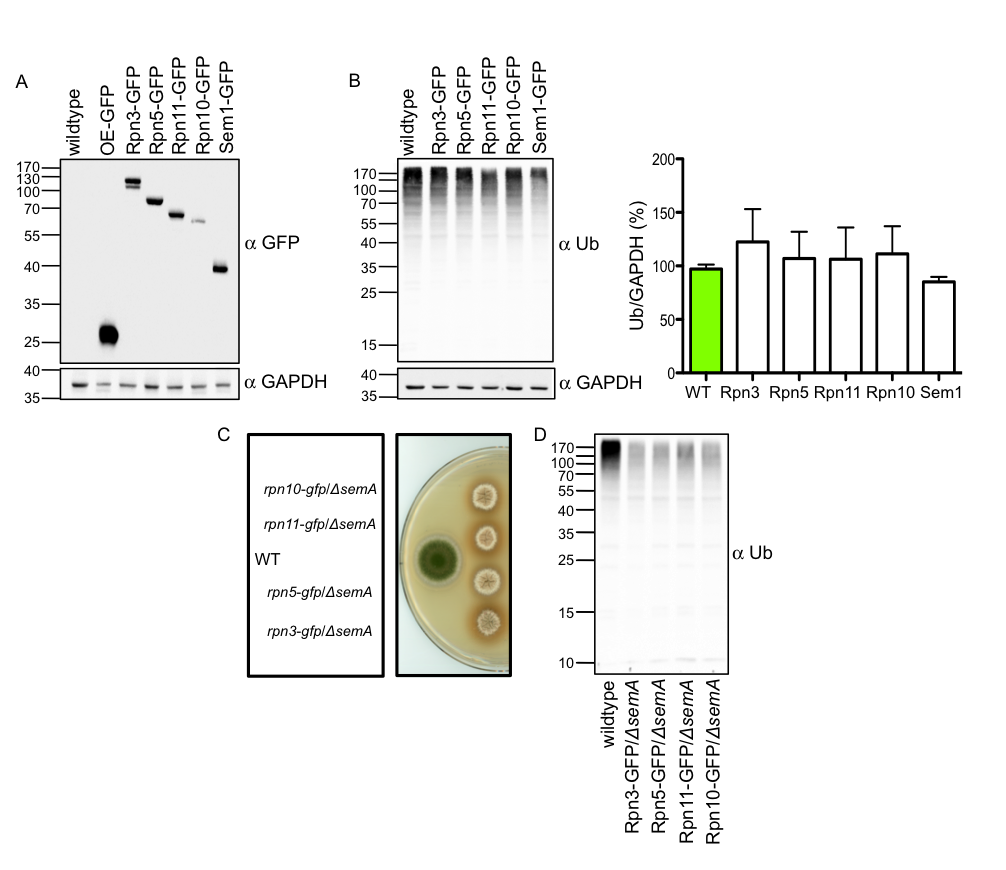


**S4 Fig. 19S RP subunits fused to GFP are functional.**

**(A)** Expression leveles of lid subunits fused to GFP in *A*. *nidulans*. The respective strains were grown vegetatively at 37°C for 20h prior to the extraction of proteins. 40μg total proteins were loaded in each lane. GFP tagged lid subunits were detected with α-GFP and glyceraldehyde-3-phosphate dehydrogenase (GAPDH) served as loading control. No GFP signal was detected in the negative control (*sem1*, wildtype), whereas the positive control (OE-GFP) showed a prominent band at the expected size of free GFP. The expected MW of the tagged proteins represent: 28.38, 99.31, 84.83, 57.94, 66.17 and 38.63 KDa, for free GFP, Rpn3-GFP, Rpn5-GFP, Rpn10-GFP, Rpn11-GFP and Sem1-GFP, respectively. **(B)** No change in total ubiquitin-conjugated substrates was observed in the GFP tagged lid subunits. Proteins were extracted after 20h of vegetative growth. Ubiqutin conjugates were detected with α-Ub and GAPDH was used as loading control. The intensity of the respective bands was determined with ImageJ v1.48 analysis software. Mean intensities from two biological replicates were normalized to the loading control GAPDH. **(C)** Asexual growth of tagged lid subunits in the absence of *sem1*. Equal numbers of spores (10,000 spores) of the respective strains were spotted on MM and grown at 37°C for 3 days in light. Top view is presented. The *sem1* strain (wildtype) showed normal asexual growth while the GFP-tagged strains lacking *sem1* showed reduced growth and accumulation of a reddish pigment, which is reminiscent to a fungal strain defective in the COP9 signalosome. **(D)** Ubiqutin-conjugates decrease in 19S RP strains lacking *sem1*. Proteins were extracted after 20h of vegetative growth. Ubiqutin conjugate proteins were detected with α-Ub (related to Fig 4).


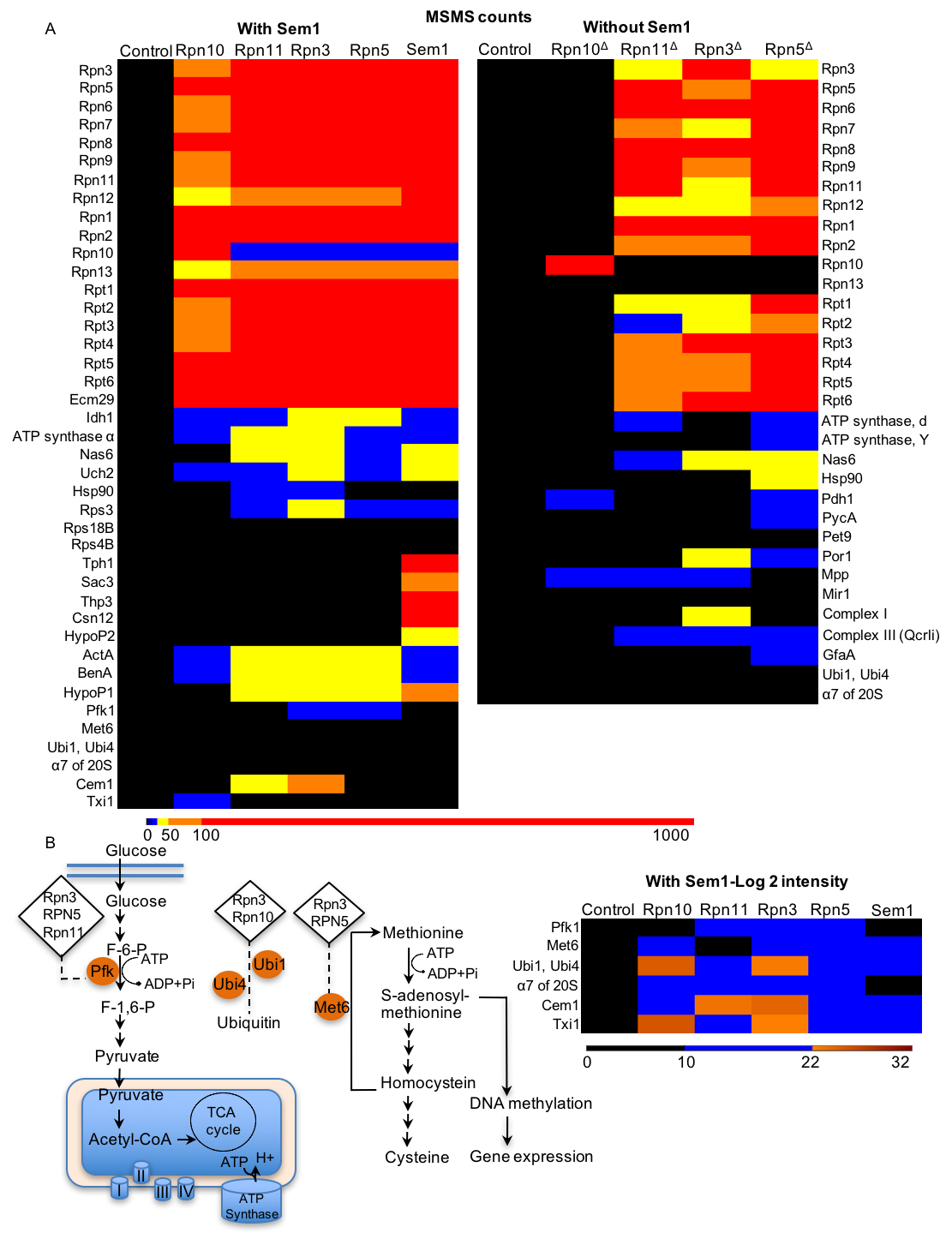


**S5 Fig. MS/MS counts of proteins associated with Sem1 and 19S RP in the presence or absence of *sem1***.

**(A)** Identified proteins were plotted according to MS/MS counts. Proteins were classified as identified, if the total number of unique peptides identified was ≥3, and the protein was present in at least two out of three biological repeats. Numbers represent the respective proteins identified in each group. Heat maps were generated by MaxQuant and plotted using Perseus. For precise log2 intensity and MS/MS counts of all proteins in this figure refer to tables S1 and S2 (related to Fig 5). Proteins in an area of low MSMS count were considered identified only if both criteria were fulfilled: LFQ>22, MS/MS counts >4. **Left panel**- 41 proteins associated with 19S rpn-gfp strains. Proteins were identified in three biological replicates plotted as heat map representing MSMS counts. **Right panel**- 33 proteins associated with 19S *rpn-gfp*::*Δsem1* strains. Each column represents the proteins identified in two biological replicates. **(B)** Rpn3, Rpn5 and Rpn10-GFP interact with proteins involved in TCA cycle, glycolysis and gene expression. Diamonds represent the indicated lid subunits; interactions are marked with doted lines. Pfk: phosphofructokinase (EC 2.7.1.11); Met6: methionine synthase (EC 2.1.1.13). Except for PFK, MetH and Ub, all other indicated enzymes were identified with SequestHT and Mascot. Note that except of Ubi1 and Ubi4, the indicated interactions were not observed when using 19S *rpn-gfp*::*Δsem1* strains (Fig 5), indicating that Sem1 mediates these associations (related to Fig 5).


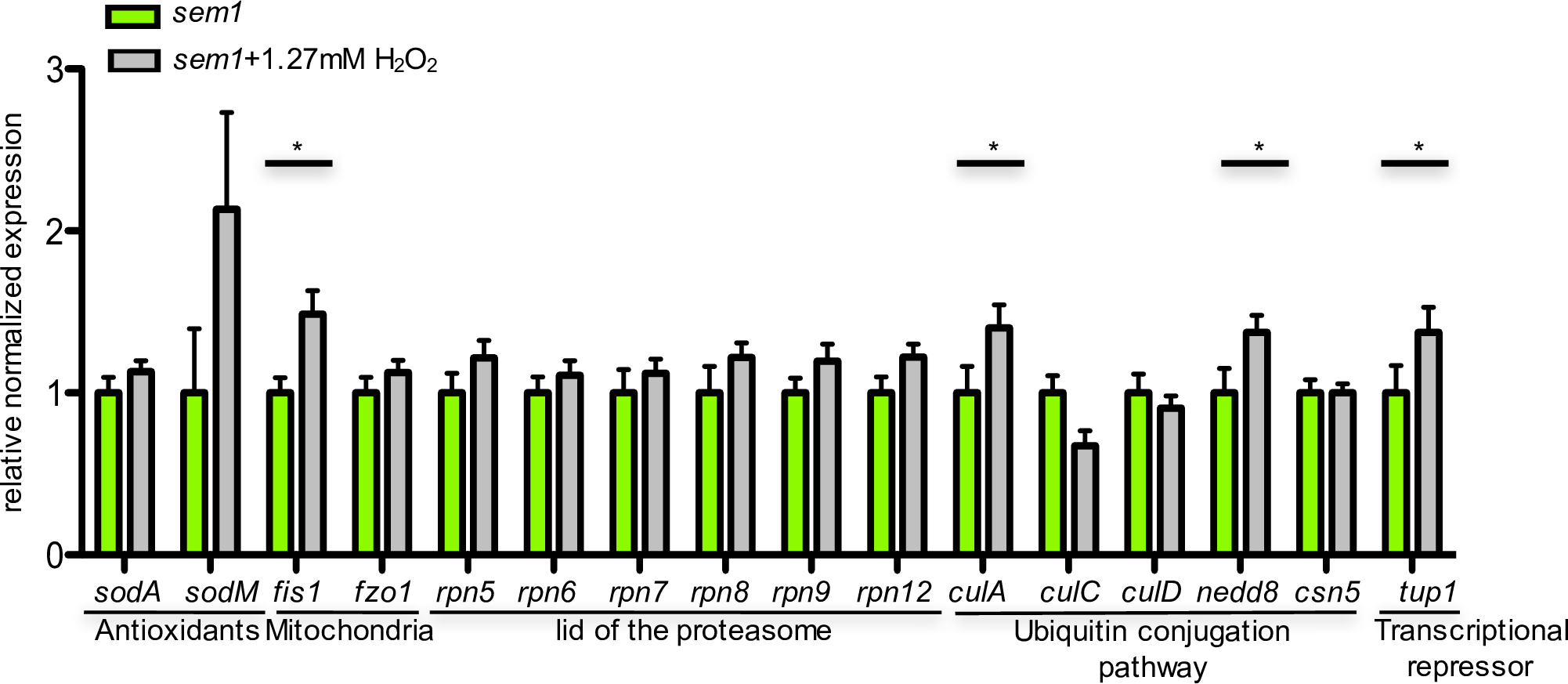


**S6 Fig. Transcript levels of superoxide dismutases (SOD), 19S RP and cullins in *sem1* exposed to 2h of oxidative stress.**

Relative expression levels after 20h of vegetative growth followed by 2h of oxidative stress (1.27mM H_2_O_2_). Bars represent mean value of four independent experiments**.** T-test of *sem1* with H_2_O_2_ vs. *sem1*, *p<0.005 (related to Fig 7).

**[2] Supplementary Tables**

**Table A. Strains used in this study**.

| Name | Genotype | Reference |
| --- | --- | --- |
| AGB551 | *nkuA*Δ*::argB*, *pyrG89*, *pyroA4*, *veA+* | [[1](#_ENREF_1)] |
| AGB653 | *nkuA*Δ, *pyrG89,* pyroA4, ^P^gpdA*::gfp::his2B* | This study |
| AGB995  **ΔsemA* | *nkuA*Δ*::argB*, *pyrG89*, *pyroA4*, *veA+*,  *semA*::*ptrA*^R^ | This study |
| *AGB996*  (**ΔsemA*::*semA*-*gfp*) | *nkuA*Δ*::argB*, *pyrG89*, *pyroA4*, *veA+*,  *ΔsemA*::*ptrA*^R^, *UTR*^semA^::*semA::gfp::nat^R^*::3'UTR*^semA^* | This study |
| AGB997  (*rpn3*-*gfp*) | *nkuA*Δ*::argB*, *pyrG89*, *pyroA4*, *veA+*, *5'UTR^rpn3^*::*rpn3::gfp::nat^R^*::3'UTR*^rpn3^* | This study |
| AGB998  (*rpn5*-*gfp*) | *nkuA*Δ*::argB*, *pyrG89*, *pyroA4*, *veA+*, *5'UTR*^rpn5^::*rpn5::gfp::nat^R^*::3'UTR*^rpn5^* | This study |
| AGB999  (*rpn10*-*gfp*) | *nkuA*Δ*::argB*, *pyrG89*, *pyroA4*, *veA+*, *5'UTR*^rpn10^::*rpn10::gfp::nat^R^*::3'UTR*^rpn10^* | This study |
| AGB1000  (*rpn11*-*gfp*) | *nkuA*Δ*::argB*, *pyrG89*, *pyroA4*, *veA+*, *5'UTR*^rpn11^::*rpn11::gfp::nat^R^*::3'UTR*^rpn11^* | This study |
| AGB1001  (**semA*-*gfp*) | *nkuA*Δ*::argB*, *pyrG89*, *pyroA4*, *veA+*, *5'UTR*^semA^::*semA::gfp::nat^R^*::3'UTR*^semA^* | This study |
| AGB1002  (*rpn3*-*gfp*:: **ΔsemA*) | *nkuA*Δ*::argB*, *pyrG89*, *pyroA4*, *veA+*, *5'UTR*^rpn3^::*rpn3::gfp::nat^R^*::3'UTR*^rpn3^*, *ΔsemA*::*ptrA*^R^ | This study |
| AGB1003  (*rpn5*-*gfp*:: **ΔsemA*) | *nkuA*Δ*::argB*, *pyrG89*, *pyroA4*, *veA+*, *5'UTR*^rpn5^::*rpn5::gfp::nat^R^*::3'UTR*^rpn5^*, *ΔsemA*::*ptrA*^R^ | This study |
| AGB1004  (*rpn10*-*gfp*:: **ΔsemA*) | *nkuA*Δ*::argB*, *pyrG89*, *pyroA4*, *veA+*, *5'UTR*^rpn10^::*rpn10::gfp::nat^R^*::3'UTR*^rpn10^*, *ΔsemA*::*ptrA*^R^ | This study |
| AGB1005  (*rpn11*-*gfp*:: **ΔsemA*) | *nkuA*Δ*::argB*, *pyrG89*, *pyroA4*, *veA+*, *5'UTR*^rpn11^::*rpn11::gfp::nat^R^*::3'UTR*^rpn11^*, *ΔsemA*::*ptrA*^R^ | This study |
| AGB1006  (*^P^TetOn-Rpn11*) | *nkuA*Δ*::argB*, *pyrG89*, *pyroA4*, *veA+*, *5'UTR*^rpn11^:: *ptrA*^R^:: *TetOn cassette: rpn11* | This study |
| AGB1068  *(***semA-yfp_c_*) | *nkuA*Δ, *pyrG89,* pyroA4, ^P^gpdA::*gfp*::*his2B,*  *niiA^T^*:: *^P^niaD*::*semA^cDNA^*:: *yfp_C_*::*niaD^T^, ptrA^R^* | This study |
| AGB1069  *(rpn10-yfp_n_*) | *nkuA*Δ, *pyrG89,* pyroA4, ^P^gpdA::*gfp*::*his2B,*  ^P^*niiA*::*rpn10^cDNA^*:: *yfp_n_*::*niiA^T^, ptrA^R^* | This study |
| AGB1070  (*rpn11-yfp_n_*) | *nkuA*Δ, *pyrG89,* pyroA4, ^P^gpdA::*gfp*::*his2B,*  ^P^*niiA*::*rpn11^cDNA^*:: *yfp_n_*::*niiA^T^, ptrA^R^* | This study |
| AGB1071  (**semA-yfp_c_+ rpn10-yfp_n_*) | *nkuA*Δ, *pyrG89,* pyroA4, ^P^gpdA::*gfp*::*his2B,*  *niiA^T^*:: *^P^niaD*::*semA^cDNA^*:: *yfp_C_*::*niaD^T^*, ::*niiA^T^,* ^P^*niiA*::*rpn10^cDNA^*:: *yfp_n_*::*niiA^T^, ptrA^R^* | This study |
| AGB1072  (**semA-yfp_c_+ rpn11-yfp_n_*) | *nkuA*Δ, *pyrG89,* pyroA4, ^P^gpdA::*gfp*::*his2B,*  *niiA^T^*:: *^P^niaD*::*semA^cDNA^*:: *yfp_C_*::*niaD^T^*, ::*niiA^T^,* ^P^*niiA*::*rpn11^cDNA^*:: *yfp_n_*::*niiA^T^, ptrA^R^* | This study |
| DH5α | *F*^−^, *Φ80dlacZ*Δ*M15*, Δ*(lacZYA*-*argF)U169*, *deoR*, *recA1*, *endA1*, *hsdR17* (rK-,mK+), *phoA*, *supE44*, *λ^−^*, *thi*-*1*, *gyrA96*, *relA1* | [[2](#_ENREF_2)] |

^P^- promoter, ^T^- terminator, ^R^- resistance

The strains were grown in minimal medium (MM) containing 1% glucose, 8.92 mM uracil, 2% AspA (70 mM NaNO_3_, 7 mM KCl, 11.2 mM KH_2_PO_4_ pH 5.5), 0.1% trace element solution, 2 mM MgSO_4_, 5 mM uridine and pyridoxine-HCl. The medium was further supplemented with appropriate antibiotics, 0.1 µg/ml pyrithiamin (ptrA for *Δsem1* [[3](#_ENREF_3)]) or 0.1 µg/ml nourseothricin (Nat, for GFP constructs [[4](#_ENREF_4)]). To prepare solid media, 2% agar was added. Plasmid DNA was propagated in DH5α grown in LB medium containing 0.1 mg/ml ampicillin at 37°C. **sem1* corresponds to *semA* in *A. nidulans* nomenclature.

**Table B. Primers used in this study.**

| Name | Sequence from: 5’ to 3’ | bp |
| --- | --- | --- |
| ****Δsem1*** | | |
| MG95 (5’.F) | CCC CTT TCA ATG AAG GGG TC | 20 |
| MG96 (Phusion.F) | CAA AAT TGC GTC CAG CGC AA | 20 |
| MG97 (5’.R) | TTA CCA ATG GGA TCC CGT AAT GGG TAC AAT TTC TCG CAA TAG A | 43 |
| MG98 (3’.F) | CTG GAT ACC CCG CCA CTC A | 20 |
| MG99 (Phusion.R) | CTG GTC TCT CCC CAC TAG C | 20 |
| MG100 (3’.R) | CAG TAT AAT ACA AAC AAA GAT GCA GCA AGA CCC CAT GGT AGC A | 42 |
| **Rpn11-GFP** | | |
| MG136 (P1) | CGG AGC AAC TCC AGA ATG CC | 20 |
| MG137 (P2) | ACT GTT GAA ACC GCA GAA CAG | 21 |
| MG138 (P3) | GCC CTT GCT CAC CAT ACC ACC GCT ACC ACC TAG TTC CTC GTC CAC ATC CAT | 51 |
| MG139 (P4) | TGA GGA TCT GAT GTC GTC TC | 20 |
| MG140 (P5) | GCT CTT GTT GGG AGG GCT TG | 20 |
| MG141 (P6) | GCT CTA CAT GAG CAT GCC CTG CCC CTG ATA GAT ATA TGC ATG AAA ATA GCT A | 52 |
| **Rpn10-GFP** | | |
| MG142 (P1) | AGG GCG AGC GGA TAG ATC TC | 20 |
| MG143 (P2) | TGT GAA GAT GGC GGC CGA G | 19 |
| MG144 (P3) | GCC CTT GCT CAC CAT ACC ACC GCT ACC ACC AGC GGT ATC CAT CTT GTC CG | 50 |
| MG145 (P4) | TGG GCT TCC TTT ATA AAG GGG | 21 |
| MG146 (P5) | CGA CTA ACT GCA TCA ATA AGT AT | 23 |
| MG147 (P6) | GCT CTA CAT GAG CAT GCC CTG CCC CTG AAT GTT TCT ATT TAA CGT TAA TGC AT | 53 |
| **Rpn5-GFP** | | |
| MG148 (P1) | GCA ACG AGC TGC AGC AAG AG | 20 |
| MG149 (P2) | TGT GCT GCG CTT TCT GAT GC | 20 |
| MG150 (P3) | GCC CTT GCT CAC CAT ACC ACC GCT ACC ACC ACG AGC CTT CGC CTT TTC CC | 50 |
| MG151 (P4) | AAT ACC AAA AAG CGT TTT CTT CA | 23 |
| MG152 (P5) | GGC ACT TGA TCG GTC CAT AC | 20 |
| MG153 (P6) | GCT CTA CAT GAG CAT GCC CTG CCC CTG ATT AAC TTC GAA AGA ACA CCA AC | 50 |
| **Rpn3-GFP** | | |
| MG154 (P1) | TTG GTT GCG GTA GCG GAG G | 19 |
| MG155 (P2) | GCG GCC GCA GCG ACA GTC AGA AAT CTA GG | 29 |
| MG156 (P3) | GCC CTT GCT CAC CAT ACC ACC GCT ACC ACC GAT GGC ATC GAA GTC CCC AC | 50 |
| MG157 (P4) | CAA AAC CAA GCA ATA CAT GCG | 21 |
| MG158 (P5) | GCG GCC GCG TAG ACA CCA AAC TGA TGT CG | 29 |
| MG159 (P6) | GCT CTA CAT GAG CAT GCC CTG CCC CTG AGG CCT GGA CCC CTG CGC | 45 |
| ***Sem1-GFP** | | |
| MG160 (P1) | CCC CTT TCA ATG AAG GGG TC | 20 |
| MG161 (P2) | CTT CTC TTC CAT CTT CAA AAT TG | 23 |
| MG162 (P3) | GCC CTT GCT CAC CAT ACC ACC GCT ACC ACC GGA TGA GGA GGC GTC GAC T | 49 |
| MG163 (P4) | CTG GAT ACC CCG CCA CTC A | 19 |
| MG164 (P5) | CAC TAG CTG TTC CTG CCC G | 19 |
| MG165 (P6) | GCT CTA CAT GAG CAT GCC CTG CCC CTG AAA CCG TGC ACA TAG ATA TGG AA | 50 |
| MG166 | GGT GGT AGC GGT GGT ATG GTG AGC | 24 |
| MG167 | TCA GGG GCA GGG CAT GCT CAT GTA | 24 |
| **Primers for RT-PCR** | | |
| **H2A (AN3468)** | | |
| MG277-RT | CGT CTT CTT CGC AAG GGA AAC T | 22 |
| MG278-RT | CGG GTT TTC TTG TTG TCA CGA G | 22 |
| **Lid subunits** | | |
| ****sem1* (AN1245)** | | |
| MG243-RT | CTG TTG ATG GTG GGT TTC CAA C | 22 |
| MG244-RT | CAG CTC TCC TCC CAC AGG TGT A | 22 |
| ***rpn3* (AN10337)** | | |
| MG323-RT | CCC GCT AAC GGT GAA AAG ACT C | 22 |
| MG324-RT | CCA TTC ACG CCA TTC TTA GTC G | 22 |
| ***rpn5 (AN4775)*** | | |
| MG325-RT | CGG TAC AAG AGA ATC CCG AAC A | 22 |
| MG326-RT | TCA ACA GGA ACA GCG GAG AGT C | 22 |
| ***rpn6 (AN10519)*** | | |
| MG327-RT | ATA CCA TGC ACT TGG CAA CCA G | 22 |
| MG328-RT | TCC AGC GAG CTG TAG CCT TCT A | 22 |
| ***rpn7 (AN1922)*** | | |
| MG329-RT | TCG AAG GCA TTC TGA ACC ACT C | 22 |
| MG330-RT | CTC CGC TTC GTC TTC TTC CTT C | 22 |
| ***rpn8 (AN5121)*** | | |
| MG331-RT | CGC AGA TCA CTA TGG ACG CTC T | 22 |
| MG332-RT | CCA CAC TGA TGG ATC CTT TTC G | 22 |
| ***rpn9 (AN3716)*** | | |
| MG333-RT | AGA TGA CCA AGA ACG GCT TTC C | 22 |
| MG334-RT | GTT CTC AAC CGC GTC AAA TGA G | 22 |
| ***rpn11 (AN4492)*** | | |
| MG245-RT | GCT GCT TTG AAA CCG GAG ACT T | 22 |
| MG246-RT | CCA CGA ATT CAC CCA GCA TAA G | 22 |
| ***rpn12 (AN3019)*** | | |
| MG335-RT | CCC GGC CTT CAC TCG ATA CTA C | 22 |
| MG336-RT | CCT TCA AGC TCG CCT CTT CAA C | 22 |
| ***rpn10 (AN7579)*** | | |
| MG337-RT | AAG GCC CAG AAG TGC TAT CCA C | 22 |
| MG338-RT | GCG GTG TTT GAG TGC AAG CTA T | 22 |
| ***rpn13 (AN1167)*** | | |
| MG339-RT | AGC TTC TGC AAG GCG AAG AGT T | 22 |
| MG340-RT | CGATTCCTGTCCCTCCTCTCTC | 22 |
| **Ubiquitin** | | |
| ***ubi1 (AN4872)*** | | |
| MG315-RT | GGT CTA CAC CAC CCC CAA GAA G | 22 |
| MG316-RT | GCC ATG AAG ATA CCA GCA CCA C | 22 |
| ***ubi4 (AN2000)*** | | |
| MG265-RT | AAA GAC GAT CAC GTT GGA GGT G | 22 |
| MG266-RT | CAT ACC ACC ACG AAG ACG AAG G | 22 |
| ***ubp6 (AN11102)*** | | |
| MG261-RT | CGG AGC ATT GAC ACG TCC TAA G | 22 |
| MG262-RT | AGA TGG GCG GTA TCT CTG AAG C | 22 |
| ***ubp14 (AN7422)*** | | |
| MG263-RT | CAA GCA CGT CAC ATT GTC CAA G | 22 |
| MG264-RT | AGG AGC CAT CAG AGT TGG AAC C | 22 |
| ***doa4 (AN2072)*** | | |
| MG259-RT | CGG AGT GGT TGG AGA AGT CAA A | 22 |
| MG260-RT | TGG GCT GGG CTG TTA TCA TAG A | 22 |
| ***rfu1 (AN3003)*** | | |
| MG257-RT | CAC CGT CTT TCT TCC ACC ACA G | 22 |
| MG258-RT | AAT CAA AAA TGG CGG ACT CGT T | 22 |
| **Ub-E3 ligases** | | |
| ***culA (AN1019)*** | | |
| MG297-RT | ACT CAT CGT CGT CCA TGG CTA A | 22 |
| MG298-RT | CCT CTC CGA GCA AGT GTG CTA A | 22 |
| ***culC (AN3939)*** | | |
| MG299-RT | GAC CAA TTT ACG GAG GCT ACG G | 22 |
| MG300-RT | TAA GCG CAA CGA CTC GAT CCT A | 22 |
| ***culD (AN10008)*** | | |
| MG301-RT | GGC GTC ATA TGT GGA GAA CAG C | 22 |
| MG302-RT | GGG CAA TCT TGT TCC CTA TTC G | 22 |
| **Denedylating enzymes and Nedd8** | | |
| ***denA (AN10456)*** | | |
| MG313-RT | TTC TGG GAG GAG TGA GTC TGG A | 22 |
| MG314-RT | GTG CGT GTG AAG TCA GGT AGG G | 22 |
| ***csn5 (AN2129)*** | | |
| MG305-RT | TAT CTC CAG TCG TGT CGG GAA G | 22 |
| MG306-RT | TGA ACG CAC CGA TGT CTA CCT T | 22 |
| ***nedd8 (AN6179)*** | | |
| MG307-RT | AGA CTC GCT ACC CTG CGT AAC C | 22 |
| MG308-RT | ACG GAG CAA GGA GAA ACG GTA A | 22 |
| **Mitochondria damage** | | |
| ***fzo1 (AN6897)*** | | |
| MG357-RT | GCT TGA GAC GTG CTG CCA CTA T | 22 |
| MG358-RT | ACC AGT TCA GCT TGC GAC AAA G | 22 |
| ***fis1 (AN6225)*** | | |
| MG359-RT | AGC CTA TAC AGC CCG TTG AAG C | 22 |
| MG360-RT | GTG GGG AAT GAA GGT GCT TAC C | 22 |
| ***Dnm1 (AN8874)*** | | |
| MG361-RT | GTG CCC AGC CCC AAC TTA CTA C | 22 |
| MG362-RT | ATT CGG GCA CCG TGG TAT AAT C | 22 |
| ***Rad23 (AN2304)*** | | |
| MG363-RT | GAA AGG GTG GGA TGT ACC TTC G | 22 |
| MG364-RT | AGG ATC TTC CCT GCA AAG TTG G | 22 |
| **Oxidative stress response** | | |
| ***atfA (AN2911)*** | | |
| MG343-RT | CAA AGT GAA CAG GCC AAA CCT G | 22 |
| MG344-RT | GGA TCC CTG AGT GGA ATC ATC C | 22 |
| ***nap1 (=yap1) (AN7513)*** | | |
| MG345-RT | CTT CAC CCA AGA CGG TCA CAT C | 22 |
| MG346-RT | GGA GGA GGG GGA ATC AGA AGT T | 22 |
| ***catA (AN8637)*** | | |
| MG347-RT | CTC TCC AAA AGA ACG GCC AGA T | 22 |
| MG348-RT | CAC ATT GCC CAG AAC TTC CTT G | 22 |
| ***sodA (AN0241)*** | | |
| MG349-RT | GCT GAT CAA GCT CAT TGG TGC T | 22 |
| MG350-RT | TCA TCA GTA CCA GCG TGA ACG A | 22 |
| ***sodB (AN5577)*** | | |
| MG351-RT | AGA GCT TCG GTA GCC TCG AGA A | 22 |
| MG352-RT | ACT ACG TCT CTG CCA CCA CGA G | 22 |
| ***sodM (AN0785)*** | | |
| MG353-RT | ACG TAA TAC CCA ATC CGC TCG T | 22 |
| MG354-RT | TCG TGA TGT ACG TTT GGT GGT G | 22 |
| ***sodE (AN1131)*** | | |
| MG355-RT | GTG TTC CGG GCA AGT TAC AGT G | 22 |
| MG356-RT | CCT TTC AAG CTC CTC GTC ATC A | 22 |
| **Primers for BiFc** | | |
| ****sem1-yfpc*** | | |
| MG365 | TCA CTT GTA CAG CTC GTC CA | 20 |
| MG366 | CGC CCG GCC TGC AAG ATC | 18 |
| MG380 | GAT CTT GCA GGC CGG GCG GGA TGA GGA GGC GTC GAC | 36 |
| MG381 | ATG TCA AAC ACT CAA TCT CAG G | 22 |
| ***Rpn10-yfpn*** | | |
| MG369 | CGC TCC ATC GCC ACG GTG A | 19 |
| MG370 | TCA CAT GAT ATA GAC GTT GTG GC | 23 |
| MG371 | ATG TCG CTT GAA GCT ACG ATG A | 22 |
| MG372 | CGT GGC GAT GGA GCG AGC GGT ATC CAT CTT GTC CG | 35 |
| ***Rpn114-yfp_n_*** | | |
| MG375 | ATG GAT AGA CTC ACT AGG ATG A | 22 |
| MG376 | CGT GGC GAT GGA GCG TAG TTC CTC GTC CAC ATC CAT | 35 |

**sem1* corresponds to *semA* in *A. nidulans* nomenclature.

**Table C. Plasmids used in this study.**

| Name | Description | Reference |
| --- | --- | --- |
| pJET | Cloning vector | Fermentas |
| pME4402 | *^P^niaD::dipA^cDNA^:: yfp_c_::niaD^T^,*  *^P^niiA::denA^cDNA^:: yfp_n_::niiA^T^;ptrA*^R^ | [[5](#_ENREF_5)] |
| pME4567 | *5’UTR^sem1^::ptrA^R^::3’UTR^sem1^* in pJET1.2 Blunt | This study |
| pME3929 | *gfp*::*nat*^R^ cassette in pJET1.2 Blunt | was kindly provided by Ö. Bayram |
| pCH008 | *ptrA^R^::^P^tpiA::rtTA2^S^M2::^T^cgrA::tetO-^P^min:: gfp* | [[6](#_ENREF_6)] |
| pME4568 | *5'UTR*^rpn3^::*rpn3::gfp::nat^R^*::3'UTR*^rpn3^*  in pJET1.2 Blunt | This study |
| pME4569 | *5'UTR^rpn5^*::*rpn5::gfp::nat^R^*::3'UTR*^rpn5^*  in pJET1.2 Blunt | This study |
| pME4570 | *5'UTR*^rpn10^::*rpn10::gfp::nat^R^*::3'UTR*^rpn10^*  in pJET1.2 Blunt | This study |
| pME4571 | *5'UTR^rpn11^*::*rpn11::gfp::nat^R^*::3'UTR*^rpn11^*  in pJET1.2 Blunt | This study |
| pME4572 | *5'UTR^sem1^*::*sem1::gfp::nat^R^*::3'UTR*^sem1^*  in pJET1.2 Blunt | This study |
| pME4573 | *nkuA*Δ*::argB*, *pyrG89*, *pyroA4*, *veA*, *5'UTR*^rpn11^:: *ptrA*^R^:: *TetOn cassette: rpn11* in pJET1.2 Blunt | This study |
| pME4617 | *niiA^T^*:: *^P^niaD*::*sem1^cDNA^*:: *yfp_C_*::*niaD^T^, ptrA^R^* in pSK409 | This study |
| pME4618 | ^P^*niiA*::*rpn10^cDNA^*:: *yfp_n_*::*niiA^T^, ptrA^R^*  in pSK409 | This study |
| pME4619 | ^P^*niiA*::*rpn11^cDNA^*:: *yfp_n_*::*niiA^T^, ptrA^R^*  in pSK409 | This study |
| pME4620 | *niiA^T^*:: *^P^niaD*::*sem1^cDNA^*:: *yfp_C_*::*niaD^T^*, ::*niiA^T^,* ^P^*niiA*::*rpn10^cDNA^*:: *yfp_n_*::*niiA^T^, ptrA^R^* in pSK409 | This study |
| pME4621 | *niiA^T^*:: *^P^niaD*::*sem1^cDNA^*:: *yfp_C_*::*niaD^T^*, ::*niiA^T^,* ^P^*niiA*::*rpn11^cDNA^*:: *yfp_n_*::*niiA^T^, ptrA^R^* in pSK409 | This study |

**Table D. Overview on data evaluation using Perseus.**


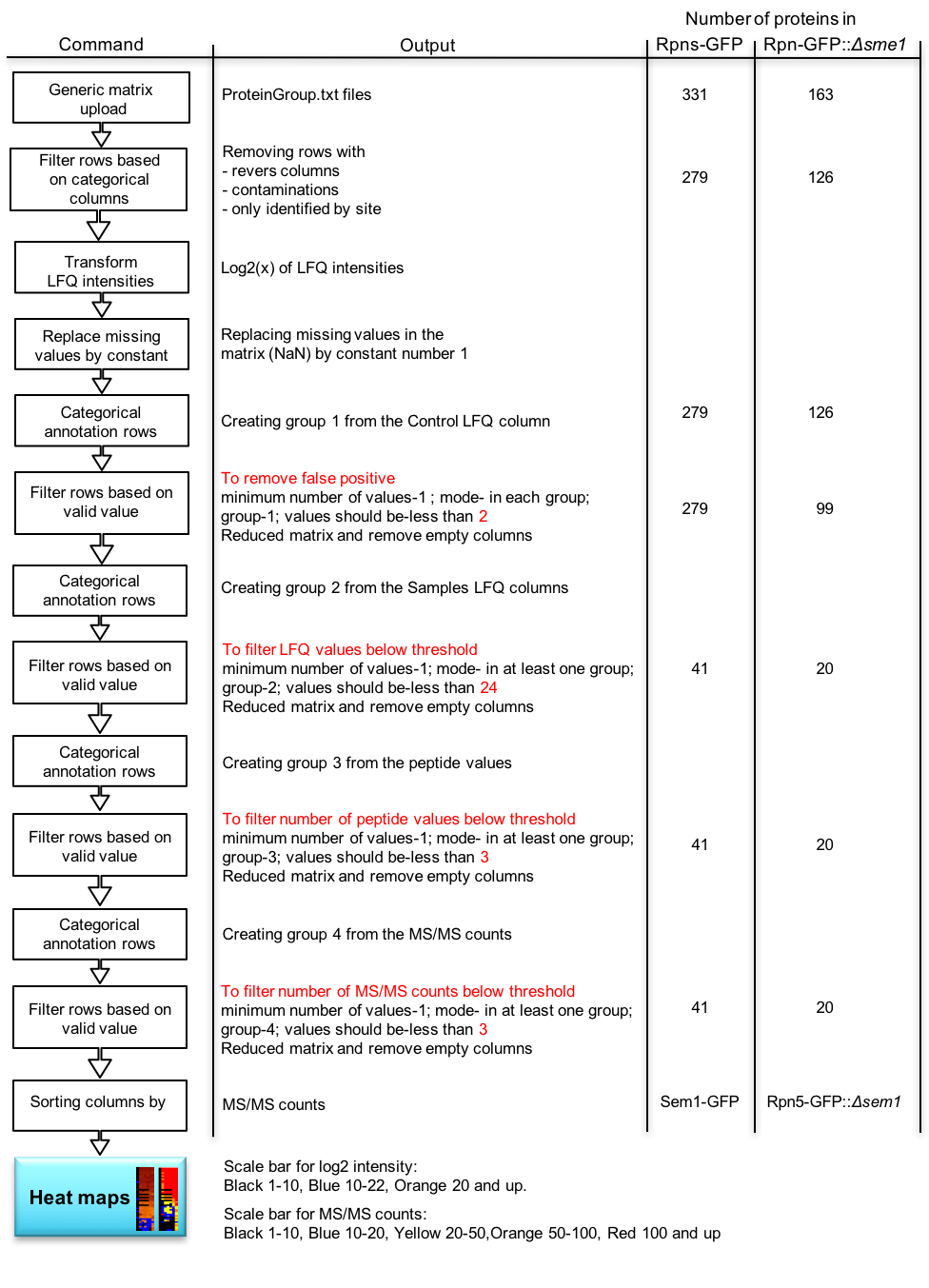


**[3] Supplementary Experimental Procedures**

**Construction of *Δsem1* strain**

A deletion cassette containing 2.0 kbp of the 5′ and 3′ flanking region of *sem1* was generated. The flanking regions were amplified from genomic DNA using the primers listed in Table B. The PCR products were ligated with the pyrithiamine (*ptrA*) resistance cassette [[7](#_ENREF_7)] using primers MG96 and MG99. Then the ligation product was cloned into linear pJET (Invitrogen) yielding plasmid pME4567. The plasmid was digested with *Hin*dIII resulting in 5712 bp-linearized fragments of *ptrA* flanked by 5’ and 3’ flanking regions of *sem1*. This linearized fragment was transformed into *A*. *nidulans* strain AGB551. Southern hybridization analysis was performed to confirm homologous recombination of the *ptrA* knockout cassette at *sem1* locus using a PCR-amplified 5’ flanking region of *sem1* as a probe.

**Construction of 19S RP-GFP strains for *in vivo* pull down**

All GFP cassettes contained (i) PCR fragment of the 5’ flanking region and the gene of interest amplified with primers P1 and P3. P3 contained addition 30 bp hanging sequence (15 bp of a linker, and 15 bp of 5’ GFP) (ii) PCR fragment of the 3’ flanking region of the gene amplified with primers P4 and P6. P6 contained additional 28bp hanging sequence identical to the 5’ region of Nat^R^ and (iii) the GFP module combined with a downstream *nat* resistance cassette [[4](#_ENREF_4)] was amplified by PCR with primers MG166/MG167 from plasmid pME3929, resulting in 2084bp fragment. The three amplified fragments were fused by PCR using primers P2 and P5 resulting in the final construct *5'UTR*^geneX^::*gfp::nat^R^*::*3'UTR^geneX^*, that was ligated into pJET1.2 resulting in pME4568, pME4569, pME4570, pME4571, pME4572, for *rpn3-gfp*, *rpn5-gfp*, *rpn10-gfp*, *rpn11-gfp* and *sem1-gfp*, respectively. The respective plasmids were propagated in DH5α. Then, the fragment of interest was either amplified from the plasmid in the case of *rpn10-gfp*, or cleaved from pJET. Subsequently the linear fragment was transformed into *A*. *nidulans*. The primers used (P1-P6) and the specific GFP cassettes in pJET are specified in Tables B and C, respectively. *In locus* recombination was confirmed by Southern hybridization.

**Construction of 19S *rpn-gfp*::*Δsem1* strains for *in vivo* pull down**

*A*. *nidulans* strains harboring *in locus* *gfp* tagged *rpn3*, *rpn5*, *rpn10* and *rpn11* were deleted for *sem1* using plasmid pME4567. *In locus* recombination was confirmed by Southern hybridization.

**Construction of BiFc strain for Sem1-Rpn10 and Sem1-Rpn11 interaction studies**

Half of a split yfp protein was fused to Sem1, Rpn10 and Rpn11 to test for interaction. Fusion proteins were under control of a bidirectional nitrate promoter. cDNA of sem1 (amplified with MG380/MG381) was fused C-terminally with a linker to the C-terminal part of yfp (yfp_c_), which was amplified from pME4402 with primers MG365/MG366. The 570 bp fragment was ligated into the SwaI restriction site of pSK409, resulting in plasmid pME4617. The N-terminal part of yfp (yfp_n_) and the appropriate linker was amplified from pME4402 using primers MG396/MG370. yfp_n_ was fused to cDNA of Rpn10 (primers MG371/MG370) and Rpn11 (Primers MG375/MG370) generating 1131 bp and 1491 bp fragments, respectively. Both fragments were ligated into the *Pme*I restriction site of pSK409 resulting in plasmids pME4618 and pME4619. *rpn10-yfp_n_ and rpn11-yfp_n_* (1131bp and 1491bp, respectively) were also ligated into pME4617 cleaved with *Pme*I to generate pME4620 and pME21 (*sem1-yfp_C_*+*rpn10-yfp_n_ and sem1-yfp_C_*+*rpn11-yfp_n_*, respectively). Plasmids pME4617, pME4618 pME4619, pME4620 and pME4621 were transformed into AGB653 resulting in strains AGB1068, AGB1069, AGB1070, AGB1071 and AGB1072, respectively. Ectopical integration was examined with Southern analyses. A probe against the nitrate promoter was amplified with primers OLKM67/OLKM68.

GFP-Trap® purification

500 ml supplemented MM in 2 liter flasks were inoculated with 5x10^5^ spores/ml and incubated at 37°C for 20h. The mycelium was filtered through Miracloth, washed three times with 0.96% NaCl solution, dried, and frozen in liquid nitrogen. Subsequently, the mycelium was grinded with the Retsch machine and transferred into 15ml falcon tubes for storage at -80°C. Two falcon tubes of the same sample were pooled into one centrifuge tube and mixed with 10ml buffer B* [(300 mM NaCl, 100 mM Tris pH 7.5, 10% glycerol, 2 mM EDTA pH=8, 0.02% NP-40) 2 mM DTT and protease inhibitor mixture from Roche (1 tablet in 500 μl water, was added before use)], followed by brief mixing and centrifugation (1h at 15,000 rpm, 4°C). The supernatant was filtered (0.2 μm filter) and added to equilibrated GFP-Trap® beads.

150 μl GFP-Trap® beads (Chromotek) were equilibrated with ice cold buffer B*. The beads were washed with 3 ml followed by additional two washing steps with 2.5 ml. The supernatant was removed by centrifugation (2 min, 2,700 x g at 4°C).

The equilibrated beads were added to filtered supernatant and the supernatant-beads mixture was incubated on a rotating platform for 2h at 4°C. The supernatant/beads mixture was poured into poly Prep chromatography column (Bio Rad) and the unbound proteins were collected. The beads were washed twice with 2.5 ml buffer ice cold W300 [300 mM NaCl, 10 mM Tris pH=7.5, 0.5 mM EDTA, 1 mM PMSF, PIM (1 tablet in 50 ml)]. Then, to remove unspecific/weak binding proteins, the beads were washed twice with 2.5 ml of ice cold W500 buffer [10 mM Tris pH=7.5, 500 mM NaCl, 0.5 mM EDTA, 1 mM PMSF, PIM (1 tablet in 50 ml), 0.02% NP-40]. Samples were stored for SDS-PAGE (wash 1-4). Elution was performed with 3x150 μl ice cold 0.2 M glycine pH=2.5 into 1.5 ml e-cap containing 9.5 μl 1 M Tris pH=10.4 to increase the final pH to 7 (elution 1-3). 50 μl of each sample was mixed with 25 μl 3xsample buffer, boiled (10 min at 95°C) and loaded on 12% SDS-PAGE.

**NanoLC-nanoESI mass spectrometry analysis and database**

NanoLC-nanoESI mass spectrometry was performed as described recently [[8](#_ENREF_8)] using peptides solutions from trypsin-digested proteins. Orbitrap-Velos raw files data were analysed with (1) Proteome Discoverer 1.4 software (Thermo Scientific, San Jose, CA, USA) using the Mascot and SequestHT search engines, or (2) with MaxQuant (<http://www.maxquant.org/>) using the Andromeda search engine. Filters for Mascot and SequestHT searches: peptide mass tolerance 10 ppm; MS/MS ion mass tolerance 0.8 Da, and up to two missed cleavages were considered. For filters used with Perseus see S4 Table. For both Proteome Discoverer and MaxQuant, methionine oxidation and carbamidomethylation were considered as variable and fixed modification, respectively. The Overview of data evaluation using Perseus is listed in table D.

**Fluorescence microscopy**

600 spores of the respective *A*. *nidulans* strains expressing GFP tagged 19S regulatory particle subunits were inoculated in 400 µl of MM+Uri+PyrHCl in an 8-well chambered cover slip (NUNC) and incubated overnight at 37°C. For cells visualization 300 µl of the media was replaced by freshly supplemented medium with 5.7 µM DAPI (Roth) and 50 nM MitoTracker Red (Molecular Probes, Invitrogen). Cells were incubated for 45 min in the dark before microscopy. Cells were visualized using Zeiss Observer. Z1 microscope equipped with CSU-X1 A1 confocal scanner unit (YOKOGAWA), QuantEM:512SC (Photometrics) digital camera and SlideBook 6.0 software package (Intelligent Imaging Innovations). Microscopy was performed using the following filters: s488G (GFP), DAPI, s561R (MitoTracker Red) with an oil immersion objective enlarging the specimen 100-fold.

**Real time PCR (RT-PCR)**

Total RNAs were extracted after 20h vegetative growth using RNeasy plant mini kit (Qiagen) and 0.8 μg RNA was transcribed into cDNA using QuantiTect reverse transcription kit (Qiagen). Expressions were measured with CFX Connect™ Real-Time System (Bio-Rad) with “MESA GREEN qPCR MasterMix Plus for SYBR® Assay” (Eurogentec). The expression of histone *h2A* was used as reference. Expression levels assayed by RT-PCR are shown as relative expression compared to wilidtype and represents mean value and standard error of the indicated independent experiments (n).

**Structure prediction**

Homology modeling of the *Aspergillus nidulans* 19S RP proteasome was performed using the comparative modeling (CM) protocol as implemented in Rosetta (RosettaCM) [[9](#_ENREF_9)]). The chosen protocol optimizes a physically realistic all-atom energy function over the conformational space defined by the structurally related template. Based on CASP10 experiments (Critical Assessment of Techniques for Protein Structure Prediction), this method of comparative modeling yields models with more accurate side-chain and backbone conformations than other methods when the sequence identity to the templates is greater than ~15%.

The initial sequence similarity search of individual proteins constituting the *A*. *nidulans* 19S RP proteasome was performed using the advanced search option available at the Protein Data Bank (PDB) web page (<http://www.rcsb.org/>220517 Supplementary MM and figures_Final.docx) [[10](#_ENREF_10), [11](#_ENREF_11)]. Based on the highest overall completeness and sequence identity to *A*. *nidulans* 19S RP proteasome (target), the cryo-EM structure of the human proteasome (EMDB 4002, PDB ids: 5L4K, 5L4G [[12](#_ENREF_12)]), resolved at 3.9 Å resolution, was used as the template for homology modeling. For each individual protein constituting the target model, the sequence alignment between the target sequence and the template sequence was obtained using PSI-Coffee, which employs an algorithm suited for alignment of distantly related proteins using homology extension [[13](#_ENREF_13)]. The homology modeling protocol (RosettaCM) required generation of a heteromeric template consisting of 18 individual proteins (sequence identity/sequence similarity, respectively): Rpn5 (44%/65%), Rpn8 (55%/71%), Rpn11 (71%/82%), Rpn3 (48%/68%), Rpn6 (49%/65%), Rpn7 (30%/46%), Rpn9 (36%/57%), Rpn12 (32%/52%), Rpn10 (47%/65%), Rpn2 (42%/61%), Rpn1 (50%/71%), Sem1 (54%/82%), Rpt1 (72%/82%), Rpt2 (67%/83%), Rpt3 (71%/84%), Rpt4 (73%/88%), Rpt5 (73%/86%), Rpt6 (77%/87%); which was used to generate several hundreds of *A*. *nidulans* 19S RP proteasome decoys. Gaps modeling or ab-initio generation of missing N- or C-terminus were not performed. The calculated decoys were energetically scored in Rosetta and subsequently clustered using Calibur [[14](#_ENREF_14)]. The core structure of the cluster with the lowest energy was selected as the final homology model of *A*. *nidulans* 19S RP proteasome.

The template structure used for homology modeling did not contain the Rpn13, which was individually modeled based on the NMR structure of the human proteasome ubiquitin receptor (PDB id 2KR0 [[15](#_ENREF_15)]) sharing 34% and 55% of the sequence identity and similarity to the target Rpn13, respectively. The modeled Rpn13 was superimposed with the Rpn13 of the yeast 26S proteasome (EMDB 3534, PDB id 5MPD [[16](#_ENREF_16)] and added to the homology model of the *A*. *nidulans* 19S RP proteasome upon structural alignment of Rpn2 proteins. Figures representing the homology model were generated with pymol (<https://sourceforge.net/projects/pymol/)>.

**[4] Supplementary References**

1. Bayram O, Bayram OS, Ahmed YL, Maruyama J, Valerius O, Rizzoli SO, et al. The *Aspergillus nidulans* MAPK module AnSte11-Ste50-Ste7-Fus3 controls development and secondary metabolism. PLoS Genet. 2012;8(7):e1002816. doi: 10.1371/journal.pgen.1002816. PMID: 22829779; PubMed Central PMCID: PMC3400554.

2. Hanahan D. Studies on transformation of *Escherichia coli* with plasmids. J Mol Biol. 1983;166(4):557-80. PMID: 6345791.

3. Kubodera T, Yamashita N, Nishimura A. Pyrithiamine resistance gene (ptrA) of *Aspergillus oryzae*: cloning, characterization and application as a dominant selectable marker for transformation. Biosci Biotechnol Biochem. 2000;64(7):1416-21. PMID: 10945258.

4. Krugel H, Fiedler G, Smith C, Baumberg S. Sequence and transcriptional analysis of the nourseothricin acetyltransferase-encoding gene nat1 from *Streptomyces noursei*. Gene. 1993;127(1):127-31. PMID: 8486278.

5. Schinke J, Kolog Gulko M, Christmann M, Valerius O, Stumpf SK, Stirz M, et al. The DenA/DEN1 Interacting Phosphatase DipA Controls Septa Positioning and Phosphorylation-Dependent Stability of Cytoplasmatic DenA/DEN1 during Fungal Development. PLoS Genet. 2016;12(3):e1005949. doi: 10.1371/journal.pgen.1005949. PMID: 27010942; PubMed Central PMCID: PMC4806917.

6. Helmschrott C, Sasse A, Samantaray S, Krappmann S, Wagener J. Upgrading fungal gene expression on demand: improved systems for doxycycline-dependent silencing in *Aspergillus fumigatus*. Appl Environ Microbiol. 2013;79(5):1751-4. doi: 10.1128/AEM.03626-12. PMID: 23275515; PubMed Central PMCID: PMC3591957.

7. Krappmann S, Jung N, Medic B, Busch S, Prade RA, Braus GH. The *Aspergillus nidulans* F-box protein GrrA links SCF activity to meiosis. Mol Microbiol. 2006;61(1):76-88. doi: 10.1111/j.1365-2958.2006.05215.x. PMID: 16824096.

8. Schmitt K, Smolinski N, Neumann P, Schmaul S, Hofer-Pretz V, Braus GH, et al. Asc1p/RACK1 Connects Ribosomes to Eukaryotic Phosphosignaling. Mol Cell Biol. 2017;37(3). doi: 10.1128/MCB.00279-16. PMID: 27821475.

9. Song Y, DiMaio F, Wang RY, Kim D, Miles C, Brunette T, et al. High-resolution comparative modeling with RosettaCM. Structure. 2013;21(10):1735-42. doi: 10.1016/j.str.2013.08.005. PMID: 24035711; PubMed Central PMCID: PMC3811137.

10. Berman HM, Westbrook J, Feng Z, Gilliland G, Bhat TN, Weissig H, et al. The Protein Data Bank. Nucleic Acids Res. 2000;28(1):235-42. PMID: 10592235; PubMed Central PMCID: PMC102472.

11. Rose PW, Prlic A, Altunkaya A, Bi C, Bradley AR, Christie CH, et al. The RCSB protein data bank: integrative view of protein, gene and 3D structural information. Nucleic Acids Res. 2017;45(D1):D271-D81. doi: 10.1093/nar/gkw1000. PMID: 27794042; PubMed Central PMCID: PMC5210513.

12. Schweitzer A, Aufderheide A, Rudack T, Beck F, Pfeifer G, Plitzko JM, et al. Structure of the human 26S proteasome at a resolution of 3.9 A. Proc Natl Acad Sci U S A. 2016;113(28):7816-21. doi: 10.1073/pnas.1608050113. PMID: 27342858; PubMed Central PMCID: PMC4948313.

13. Di Tommaso P, Moretti S, Xenarios I, Orobitg M, Montanyola A, Chang JM, et al. T-Coffee: a web server for the multiple sequence alignment of protein and RNA sequences using structural information and homology extension. Nucleic Acids Res. 2011;39(Web Server issue):W13-7. doi: 10.1093/nar/gkr245. PMID: 21558174; PubMed Central PMCID: PMC3125728.

14. Li SC, Ng YK. Calibur: a tool for clustering large numbers of protein decoys. BMC Bioinformatics. 2010;11:25. doi: 10.1186/1471-2105-11-25. PMID: 20070892; PubMed Central PMCID: PMC2881085.

15. Chen X, Lee BH, Finley D, Walters KJ. Structure of proteasome ubiquitin receptor hRpn13 and its activation by the scaffolding protein hRpn2. Mol Cell. 2010;38(3):404-15. doi: 10.1016/j.molcel.2010.04.019. PMID: 20471946; PubMed Central PMCID: PMC2887722.

16. Wehmer M, Rudack T, Beck F, Aufderheide A, Pfeifer G, Plitzko JM, et al. Structural insights into the functional cycle of the ATPase module of the 26S proteasome. Proc Natl Acad Sci U S A. 2017;114(6):1305-10. doi: 10.1073/pnas.1621129114. PMID: 28115689; PubMed Central PMCID: PMC5307450.
